# Comparison of Metformin Combinations with Other Repurposed Drugs in the Treatment of Hamster Fibrosarcoma: A Review

**DOI:** 10.3390/ph19081254

**Published:** 2026-08-09

**Authors:** Dušica J. Popović, Kosta J. Popović, Dejan Miljković, Mihalj Poša, Zana Dolićanin, Ivan Čapo, Jovan K. Popović

**Affiliations:** 1 Department of Biomedical Sciences, State University of Novi Pazar, Vuka Karadžića 9, 36300 Novi Pazar, Serbia; dusicapopovic@np.ac.rs (D.J.P.); zdolicanin@np.ac.rs (Z.D.); 2 Department of Pharmacy, Faculty of Medicine, University of Novi Sad, Hajduk Veljkova 3, 21000 Novi Sad, Serbia; kosta.popovic@mf.uns.ac.rs (K.J.P.); mihalj.posa@mf.uns.ac.rs (M.P.); 3 Department of Histology and Embryology, Faculty of Medicine, University of Novi Sad, Hajduk Veljkova 3, 21000 Novi Sad, Serbia; dejan.miljkovic@mf.uns.ac.rs (D.M.); ivan.capo@mf.uns.ac.rs (I.Č.); 4 Department of Pharmacology, Toxicology and Clinical Pharmacology, Faculty of Medicine, University of Novi Sad, Hajduk Veljkova 3, 21000 Novi Sad, Serbia; 5 Academy of Medical Sciences of the Serbian Medical Society, 19 George Washington Str., 11000 Belgrade, Serbia

**Keywords:** fibrosarcoma, hamsters, metformin, disulfiram, diclofenac, nitroglycerin, itraconazole, caffeine

## Abstract

**Background/Objectives:** Metformin is a prominent candidate for cancer drug repurposing, backed by preclinical and epidemiological evidence showing reduced cancer incidence in diabetic patients. Its pleiotropic effects include AMPK activation, protein synthesis inhibition, and metabolic alterations. This review integrates global preclinical data via a comprehensive tabular overview alongside a cross-analysis of specific investigations of hamster fibrosarcoma. Since head-to-head comparisons of metformin-based combinations on hamster fibrosarcoma remain limited, this work performs an integrated cross-study evaluation to establish a clear comparative hierarchy of various repurposed adjuvants combined with metformin. **Methods:** A literature review and cross-study re-analysis of peer-reviewed preclinical studies were conducted, focusing on in vivo therapeutic outcomes within the BHK-21/C13-induced hamster fibrosarcoma model. Treatment regimens from distinct primary studies were evaluated side-by-side using tumor endpoint data expressed as a percentage of control mean (± SD). Only statistically significant pairwise differences (*p* < 0.05) determined the comparative hierarchy. **Results:** Integrated analysis identified disulfiram and diclofenac as the most promising adjuvants among compared metformin combinations, showing the highest statistical significance across all evaluated endpoints. The statistical ranking of the metformin-based combinations followed a descending order: disulfiram, diclofenac, nitroglycerin, itraconazole, and caffeine. **Conclusions:** By synthesizing previously fragmented primary data into a unified comparative framework, these findings provide a strong, consolidated preclinical rationale for metformin-based combination strategies. The established hierarchy of adjuvant potency resolves structural ambiguity and supports further translational and clinical investigation to evaluate their therapeutic potential in fibrosarcoma and other malignancies.

## 1. Introduction

Galega officinalis (goat’s rue; *Herba rutae caprariae*) is a historically significant medicinal plant that served as the natural precursor for the synthesis of biguanides in the early 20th century. Metformin (1,1-dimethylbiguanide), first synthesized in 1922 by Werner and Bell at Trinity College Dublin, remains the most prominent derivative. The plant has a long record of medicinal use, with one of the earliest detailed botanical descriptions provided in 1772 by John Hill. Traditionally, goat’s rue was employed to alleviate symptoms associated with diabetes, including polydipsia and polyuria, and was also used in the management of infectious diseases such as smallpox and measles, as well as for treating venomous bites. In addition to its therapeutic applications, it was utilized in traditional dairy production in Mediterranean cultures [1].

In 1957, the antihyperglycaemic efficacy of biguanides was clinically demonstrated in human studies conducted in France. Since its broader approval in 1995, metformin (marketed as Glucophage) has become one of the most widely prescribed glucose-lowering agents worldwide.

Initial experimental evidence for the anticancer activity of metformin was reported in animal studies in 2001 [2], followed by retrospective observational analyses indicating an association between metformin use and reduced cancer risk in patients with type 2 diabetes.

The first clinical repurposing of metformin for cancer prevention was reported in 2005 through an observational case–control study conducted in Tayside, involving approximately 11,000 patients with type 2 diabetes. The analysis showed that metformin use was associated with a 23% reduction in overall cancer risk, based on comparisons between 923 patients with cancer and 1,846 controls. These findings provided early evidence supporting a potential protective role of metformin against cancer. Subsequent observational studies using clinical and insurance databases worldwide further explored this association [3].

Since 2005, studies have reported a reduced cancer risk in patients with diabetes [4]. Metformin has become a key focus of drug repurposing research due to its multifaceted mechanisms, including enhanced insulin sensitivity, modulation of glucose uptake, effects on gluconeogenesis and protein synthesis, and gut microbiome alterations, as well as direct activation of AMP-activated protein kinase (AMPK). These actions contribute to decreased DNA damage, improved DNA repair, induction of autophagy, inhibition of cell growth, and suppression of proliferation and neoangiogenesis [5].

Following the establishment of metformin effects on glycemic control and insulin sensitivity, numerous preclinical studies have investigated the mechanisms underlying its anticancer activity (Table A1 [2,6,7,8,9,10,11,12,13,14,15,16,17,18,19,20,21,22,23,24,25,26,27,28,29,30,31,32,33,34,35,36,37,38,39,40,41,42,43,44,45,46,47,48,49,50,51,52,53,54,55,56,57,58,59,60,61,62,63,64,65,66,67,68,69,70,71,72,73,74,75,76,77,78,79,80,81,82,83,84,85,86,87,88,89,90,91,92,93,94,95,96,97,98,99,100,101,102,103,104,105,106,107,108,109,110,111,112], Appendix A, and Table 1). Given the extensive volume of the literature surrounding metformin, a targeted search strategy was employed to compile the data for the tables. A search of the PubMed database using the keywords ‘metformin’ and ‘cancer’ within the Title/Abstract fields yielded approximately 5490 articles, while a complementary search in the ScienceDirect database using the same keyword combination retrieved 1655 results. To maintain a strict focus on basic and translational mechanisms, the retrieved records were manually screened based on predefined criteria. Only primary research articles possessing a registered DOI and evaluating preclinical (in vitro and in vivo) oncological outcomes were selected. To preserve the preclinical scope of Table 1 and Appendix A Table A1, review articles and clinical trials were excluded from these specific datasets. The vast majority of the cited functional studies in this review are indexed in PubMed and were chosen to reflect representative mechanistic pathways and combination strategies over the last two decades. In addition to its metabolic actions, metformin modulates key signaling pathways that regulate cell growth and survival. These mechanisms may be broadly classified as insulin-dependent and insulin-independent. A key pathway involves activation of AMPK, leading to inhibition of mTOR and consequent cell cycle arrest, apoptosis, and suppression of tumor growth. Metformin also modulates major oncogenic pathways, including PI3K/Akt, Erk, and RTKs, while affecting tumor metabolism, angiogenesis, and immune responses. Furthermore, inhibition of mitochondrial complex I disrupts cellular bioenergetics and the Warburg effect, reinforcing its anticancer potential.

Table A1 [2,6,7,8,9,10,11,12,13,14,15,16,17,18,19,20,21,22,23,24,25,26,27,28,29,30,31,32,33,34,35,36,37,38,39,40,41,42,43,44,45,46,47,48,49,50,51,52,53,54,55,56,57,58,59,60,61,62,63,64,65,66,67,68,69,70,71,72,73,74,75,76,77,78,79,80,81,82,83,84,85,86,87,88,89,90,91,92,93,94,95,96,97,98,99,100,101,102,103,104,105,106,107,108,109,110,111,112] (Appendix A) provides a detailed summary of preclinical studies reporting metformin’s anticancer activity, either alone or in combination, across various cancer types since 2001. Table 1 offers a concise overview of studies highlighting the anticancer effects of metformin specifically in combination with other drugs or treatments. In these combinations, metformin enhances sensitivity to, or reduces resistance against, the co-administered agent.

When analyzing the data compiled in Table 1 and Appendix A Table A1, several limitations in the current literature must be explicitly acknowledged. First, some preclinical in vitro evidence relies on clinically unrealistic metformin concentrations. Consequently, some of the proposed molecular mechanisms observed in laboratory settings may not translate effectively to human physiology. Furthermore, a portion of the comparative evidence regarding specific metformin combinations originates from research groups with overlapping authorship. While these studies provide foundational insights, further independent replication will be valuable to support the broader translational scalability of these findings.

This review aims to highlight the vast and ever-expanding volume of preclinical research dedicated to investigating the anticancer properties of metformin over the last two decades. To address this extensive scientific backdrop, a comprehensive tabular overview encompassing over 100 relevant primary references has been compiled, integrating worldwide preclinical data alongside specific in vivo investigations of hamster fibrosarcoma (Table 1 and Appendix A Table A1). Building upon this context, the primary purpose of this review is to systematically compare and synthesize preclinical data regarding the anticancer efficacy of metformin, both alone and in combination with various repurposed agents, specifically within the BHK-21/C13-induced hamster fibrosarcoma model. This review aims to bridge the gap between individual primary studies [45,46,47,48,49,50] by performing an integrated cross-study evaluation. By evaluating these distinct combination strategies side-by-side, this work establishes a comparative hierarchical ranking of repurposed adjuvants, specifically disulfiram, diclofenac, nitroglycerin, itraconazole, and caffeine, based on their therapeutic outcomes and statistical significance. Ultimately, this comprehensive synthesis serves to uncover possible overarching synergistic mechanisms and provide a consolidated preclinical rationale required to guide future clinical investigations in the treatment of fibrosarcoma and other types of cancer.

While most preclinical studies show promising anticancer effects of metformin, clinical results have been variable. Despite strong preclinical and epidemiological evidence, clinical outcomes of metformin in oncology remain inconsistent, likely reflecting limitations in study design, patient populations, and dosing regimens. Future studies are needed to clarify the complex interactions between metabolic regulation and oncogenic signaling and to fully exploit metformin as a safe, accessible, and cost-effective adjunct therapy [113,114].

## 2. Metformin and Its Combinations in the Treatment of Hamster Fibrosarcoma

Since 2017, a series of experiments has been conducted to investigate the anticancer effects of metformin, both as a monotherapy and in various combinations with other repurposed drugs, on hamster fibrosarcoma in human-equivalent doses (Table 2, Table 3, Table 4, Table 5 and Table 6).

All animal experiments were conducted using the methodology described in our previous studies [45,46,47,48,49,50,51,52]. Syrian golden hamsters were randomly assigned to experimental groups of minimum 6 animals of comparable age and body weight in each experiment. Animals were housed under standard laboratory conditions, and all procedures were approved by the University of Novi Sad Animal Ethics Committee (Novi Sad, Serbia). Sex-related differences and the potential influence of sex hormones on tumor growth were not evaluated. In accordance with the 3R principles, the number of animals per group was minimized while maintaining statistical relevance.

Syrian hamsters were selected because the BHK-21/C13 cell-induced sarcoma model is highly reproducible, consistently producing a solitary, locally invasive tumor at the inoculation site without detectable metastasis. The resulting tumors closely resemble BHK-21/C13 cells grown in vitro, as hamster immune mechanisms do not recognize these cells as tumorigenic, thereby preventing immune-mediated rejection and allowing extensive tumor growth. Since only viable BHK-21/C13 cells are tumorigenic, the resulting BHK tumor can be considered an in vivo counterpart of the original cell culture. Owing to the absence of significant immune interference, this model provides a suitable platform for the pharmacological evaluation of anticancer agents. BHK-21/C13 cells were subcutaneously inoculated into the dorsal region of each hamster (1 mL, 2x10^6^ cells/mL) by the same investigator.

Human-to-animal dose extrapolation for the repurposed drugs was performed via body surface area (BSA) normalization, adhering to the US FDA guidance (*Estimating the Maximum Safe Starting Dose in Initial Clinical Trials for Therapeutics in Adult Healthy Volunteers*) criteria. Accordingly, hamster doses corresponded to standard human therapeutic doses expressed in mg/kg/day and adjusted using BSA conversion factors [45,46,47,48,49,50]:

HED (mg/kg) = Animal dose (mg/kg) × [Animal weight (kg)Human weight (kg)] ^(1−EXP)^ (1)

where HED is the human-equivalent dose and EXP is an allometric scaling exponent. The FDA guidance used conversion factors that were derived based on a BSA scaling factor of 0.67, i.e., (1 − EXP) = 1 − 0.67 = 0.33.

BSA scaling alone does not guarantee pharmacokinetic or pharmacodynamic equivalence across species. Species-specific differences in drug absorption, first-pass metabolism, half-life, active metabolites, tissue distribution, and tolerated exposure can drastically alter drug activity. Consequently, the absence of plasma or intratumoral concentration measurements may represent a translational limitation of this study.

Following tumor cell inoculation, all drugs were administered orally once daily by gastric gavage as either solutions (metformin) or suspensions in saline. Individual doses were adjusted to body weight to maintain equivalent mg/kg exposure, whereas the gavage volume (approximately 1 mL) was based on standard hamster fluid intake and remained substantially below normal daily consumption levels.

To ensure animal welfare, in all experiments humane endpoints were strictly defined as a ≥ 20% loss in body weight, reduced responsiveness, postural impairment, an inability to maintain normal physiological functions (eating, urination, or defecation), tumor diameter >3.5 cm, tumor burden exceeding 10% of body weight, and tumor ulceration. Animals were monitored daily for body weight, behavior, general condition, clinical signs (including diarrhea, neurological symptoms, and respiratory disturbances), as well as tumor size, location, multiplicity, and ulceration.

At the end of each experiment, hamsters were euthanized via an intraperitoneal injection of pentobarbital (90 mg/kg). Deep anesthesia and loss of consciousness were verified after 5 min by the absence of spontaneous respiration and the lack of pedal and digital reflex responses. Following confirmation, terminal cardiac exsanguination (3.0–5.5 mL) was performed to collect blood samples for biochemical and hematological analyses. Subsequently, major vital organs, including the brain, heart, lungs, liver, kidneys, stomach, and intestines, were collected postmortem for downstream pathological, histological, and toxicological evaluations.

Throughout the experimental period, hamster body weight and tumor dimensions were monitored daily using digital calipers, with tumor volume calculated via the standard ellipsoid formula. Animal welfare remained optimal across all cohorts; no premature mortality or unplanned euthanasia occurred. Maximum tumor diameters remained strictly below 3.5 cm, and the tumor burden remained well below the 10% threshold of total body mass, thereby complying with international ethical guidelines across all experiments. Following surgical excision, tumors were weighed, and their three-dimensional axes were measured to calculate surface area using the ellipsoid model. Precise tumor volume was determined through the standard water displacement method. For subsequent histopathological and immunohistochemical evaluations, representative cross-sections (up to 5 mm thick) were harvested from the maximum circumference of each tumor nodule.

Standard hematoxylin–eosin (HE) staining was performed to evaluate tumor growth, tissue infiltration, and the areas of necrosis and hemorrhage. To complement the histopathological assessment, immunohistochemical (IHC) staining was carried out using a panel of biomarkers to evaluate specific cellular processes: Ki-67 and PCNA for cellular proliferation; CD34 and CD31 for neoangiogenesis; GLUT1 for glucose metabolism; iNOS for nitric oxide pathways; and COX4 alongside Cytochrome C to monitor apoptosis.

In all experiments, quantitative data were expressed as mean ± standard deviation (SD) or standard error (SE). Inter-group variations were evaluated using one-way analysis of variance (ANOVA) followed by the Student–Newman–Keuls post hoc test, utilizing TIBCO Statistica 13.3.1 software (TIBCO Software, Inc.). To validate parametric assumptions, the two-sided Mann–Whitney U test was additionally applied for median comparisons. Correlation analysis was also performed where appropriate. In all statistical evaluations, a *p*-value less than 0.05 was considered to indicate statistical significance.

Across all experiments, the evaluated treatments demonstrated a favorable safety profile, exerting no significant effects (*p* > 0.05) on animal body weight, hematological parameters (erythrocyte, leukocyte, and platelet counts; hemoglobin and hematocrit levels; erythrocyte sedimentation rate), or biochemical markers (glucose and serum protein levels) compared to the control groups.

In this review, the anticancer efficacy of metformin [45] and its combinations with caffeine [46], itraconazole [47], nitroglycerin [48], disulfiram [49], and diclofenac [50] was comparatively evaluated based on control-versus-treatment *p* values and the percentage change in the measured endpoints relative to the corresponding control groups.

## 3. Comparative Assessment of Metformin-Based Treatments Against Hamster Fibrosarcoma

To facilitate a comparative assessment of metformin-based treatments, we: (1) evaluated the 95% confidence intervals for the primary endpoints (Table 7), and (2) ranked statistically significant treatments across multiple tumor-related endpoints (Table 8 and Table 9). The ranking approach was based on ascending *p* values, with lower *p* values indicating stronger statistical evidence of treatment-associated changes. Separate rankings were generated for macroscopic tumor characteristics and immunohistochemical markers to provide an integrated overview of treatment performance across distinct biological domains. The resulting rankings are presented in Table 8 and Table 9.

Table 7 presents the 95% confidence intervals (95% CI) and interval widths for the primary study endpoints (tumor weight and tumor volume) and the secondary endpoint (Ki-67 expression) across the 29 evaluated experimental groups. Statistical significance (where the 95% CI does not include zero) was consistently achieved in several distinct experimental subsets. Specifically, Experiments 1, 4, 5, 6, 9, 10, 12, 15, and 17 demonstrated robust and statistically significant anticancer efficacy, as evidenced by strictly positive confidence intervals across all analysed endpoints in Table 7. Conversely, the remaining experimental groups (2, 3, 7, 8, 11, 13, 14, 16, 18, 19, 20, 21, 22, 23, 24, 25, 26, 27, 28, and 29) exhibited wider confidence intervals that spanned across zero, which indicates a higher degree of data variance and a lack of clear statistical significance under those specific dosing, dual-combination or rescue three-drug combination protocols. Notably, the tightest and most consistent confidence intervals for the primary endpoints were observed in Experiments 17 (Met 500 + Dic 60), 15 (Met 500 + Dis 50), 1 (Met 500, pretreatment 7 days + treatment 14 days), and 5 (Met 500 + Itra 250), underscoring the reproducible therapeutic potential of these specific intervention regimes. It should be noted that a regimen beginning seven days before inoculation (pretreatment) has different clinical implications from therapeutic administration after tumor establishment.

## 4. Comparative Analysis of Metformin and Metformin-Based Combination Treatments with Significant Anticancer Activity

Following the identification of metformin-based treatment regimens with significant anticancer activity across multiple tumor endpoints, pairwise comparisons were performed to further evaluate differences among the most effective treatments. The analysis was based on tumor endpoint values expressed as percentages of the control mean (± SD), enabling direct comparison of treatment-associated effects across the evaluated parameters. Only statistically significant differences (*p* < 0.05) between treatment pairs were retained and are summarized in Table 10.

Our comparisons rely on published summary statistics [45,46,47,48,49,50]. To ensure consistency, data normalization was executed relative to the control groups reported in the analyzed publications [45,46,47,48,49,50].

Our analyses indicate that the disulfiram–metformin and diclofenac–metformin combinations possess the narrowest 95% CI widths for the primary endpoints (Table 7) and were included in the majority of drug combinations exhibiting the most statistically significant anticancer effects, as reflected by the lowest *p* values (Table 8 and Table 9) across all evaluated endpoints.

Furthermore, disulfiram was among the most frequently co-administered agents in the most effective treatment regimens identified through comparisons with other effective therapeutic approaches (Table 10). Following disulfiram, the remaining drugs were ranked in descending order of efficacy as diclofenac, nitroglycerin, itraconazole, and caffeine (Table 8, Table 9 and Table 10).

Within the evaluated studies [45,46,47,48,49,50], monotherapies generally failed to elicit any anticancer response, with the notable exception of metformin under specific experimental conditions. Metformin monotherapy demonstrated antineoplastic efficacy exclusively when administered prophylactically prior to tumor inoculation or at elevated doses. This pattern was evident in the following therapeutic regimens (experiments numbered in Table 2): (1) 500 mg/kg/day of metformin involving a 7-day pretreatment phase followed by a 14-day post-inoculation course (Table 8 and Table 9); (7) 500 mg/kg/day of metformin with a 3-day pretreatment and a 14-day post-inoculation treatment (Table 8); and (16) 1000 mg/kg/day of metformin administered for 19 days post-tumor inoculation (Table 9), consistent with evidence from epidemiological studies on long-term antidiabetic metformin therapy [3,4].

## 5. General Discussion and Conclusions

Overall, in our experiments, the metformin-based combinations produced statistically significant and potentially synergistic inhibition of hamster fibrosarcoma growth (Table 7, Table 8 and Table 9). These findings were further supported by additional validation experiments, including NF-κB activation-based rescue approaches and complementary in vitro studies in multiple malignant cell lines. Specifically, co-administration of an NF-κB stimulator (e.g., mebendazole, experiments 28 and 29, Table 2, Table 3, Table 4, Table 5 and Table 6) completely abolished the combined antitumor action of metformin and the co-repurposed drugs, fully rescuing tumor progression [48,49,52].

However, the mebendazole rescue experiments do not establish NF-κB as the exclusive causal mechanism, because mebendazole also affects microtubules, cellular stress, metabolism, and other pathways. The loss of antitumor activity may therefore reflect off-target effects, pharmacokinetic interactions, toxicity, or pathway crosstalk. Stronger mechanistic claims require direct NF-κB target-engagement data, such as p65 nuclear localization, transcriptional activity, phosphorylation, downstream gene expression, and appropriate genetic or pharmacological controls.

The observed anticancer activity of the metformin-based combinations is likely attributable to the potentially synergistic modulation of NF-κB signaling, acting at upstream, downstream, or direct regulatory levels. Such combination strategies may enhance the therapeutic effects of individual agents, with potential relevance for fibrosarcoma and other malignancies in chemopreventive or adjuvant settings. Potential advantages include increased antitumor efficacy, dose reduction in individual compounds (thereby limiting adverse effects), and possible circumvention of drug resistance.

Since drug concentrations were not determined, the original Combination Index (CI) equation [115] was adapted by substituting drug concentrations with administered doses, assuming linear pharmacokinetics in which dose is proportional to drug concentration in body fluids. The dose-modified Combination Index (CI) was calculated using the adapted equation:

CI = C_A_/IC_A_ + C_B_/IC_B_ ≈ D_A_/ID_A_ + D_B_/ID_B_ (2)

where IC (or ID) is the concentration (dose) of a single agent required to achieve a given effect, and C (or D) is the concentration (dose) of each drug in the combination producing the same effect. In this study, D represents the dose of metformin (A) or the second repurposed drug (B) in combination that produced a statistically significant improvement over the control for the evaluated endpoints (e.g., tumor weight, volume, burden, Ki-67 expression). As neither monotherapy produced significant effects, whereas all combinations were highly effective at the tested doses, the calculated CI values remained < 1, indicating potentially synergistic drug interactions.

In the rescue experiments, conducted to elucidate the underlying, possibly synergistic mechanisms, the intended outcome of the metformin-based two-drug combinations supplemented with NF-κB stimulator mebendazole was preservation of tumor weight, volume, burden and other defined endpoints at levels comparable to the control, with no statistically significant differences across all evaluated endpoints. The CI was calculated using the modified Chou–Talalay equation:

CI = C_A_/IC_A_ + C_B_/IC_B_ + C_C_/IC_C_ ≈ D_A_/ID_A_ + D_B_/ID_B_ + D_C_/ID_C_ (3)

where subscript A represents metformin, B the second repurposed drug, and C mebendazole. For the three-drug combinations, the combination index (CI) for metformin-based regimens supplemented with mebendazole was calculated using human-equivalent doses. This calculation was performed assuming zero intrinsic anticancer activity for mebendazole, a premise that was confirmed experimentally in the experimental groups receiving mebendazole as a monotherapy [48,49,52]. Due to the absence of single-agent anticancer activity of mebendazole in this rescue model, its inclusion in the modified Chou–Talalay equation yielded combination index CI values > 1. This suggests potential antagonistic interactions. Consequently, the addition of mebendazole abolished the antitumor efficacy of the two-drug metformin combinations, showing no statistically significant differences from the control group across all evaluated endpoints. Consistent with this model, all calculated values exceeded 1 (CI > 1), suggesting a potential antagonistic anticancer effect underpinned by NF-κB modulation.

A limitation of this CI analysis is the use of administered doses instead of measured plasma or tissue drug concentrations. The approach assumes linear pharmacokinetics and may not fully account for in vivo variability, including nonlinear kinetics, saturable absorption, or tissue-specific drug distribution. Since our data lacked the required dose–response relationships and uncertainty estimates necessary for a robust model, the results of this modified Chou–Talalay analysis should be accepted solely under the aforementioned limitations.

The enhanced antitumor activity observed with combination treatment suggests potential synergistic interactions; however, this conclusion remains hypothetical and preliminary due to the limitations of the in vivo model and the lack of comprehensive pharmacokinetic data. The CI analysis was based on tumor growth endpoints rather than measured drug concentrations, and the relatively small sample size may have reduced statistical precision. In addition, biological variability and tumor microenvironmental factors inherent to in vivo models are not fully captured by conventional synergy analyses. Therefore, these findings should be considered exploratory, and future studies incorporating direct pharmacokinetic measurements are needed to validate the preliminary estimated CI values.

Nevertheless, these findings should be interpreted as preliminary preclinical evidence. Before any clinical translation, further studies are required, including detailed mechanistic validation, pharmacokinetic profiling, confirmation of systemic and intratumoral exposure, evaluation across multiple tumor models, and assessment of safety margins.

Gross pathological and histopathological changes were not detected throughout the analyzed studies [45,46,47,48,49,50]. Furthermore, no drug–drug toxicity was observed in the tested hamsters; however, the small sample size (six to eight animals per group) leaves the study underpowered to detect moderate or subtle toxicities. Consequently, stable body weights and limited parameters cannot conclusively establish safety [45,46,47,48,49,50]. All utilized drugs are currently registered and in clinical use. While human toxicity at standard therapeutic doses is not expected, definitive drug–drug toxicity profiles in humans must be further evaluated through prospective clinical trials in patient cohorts.

Metformin exerts pleiotropic anticancer effects through modulation of several signaling pathways. AMPK activation inhibits mTOR signaling, thereby inducing apoptosis, autophagy, and cell cycle arrest while suppressing protein synthesis, cell proliferation, migration, and invasion. Furthermore, metformin inhibits MAPK signaling, promotes apoptosis, and limits cell migration. Its ability to suppress NF-κB signaling has also been linked to reduced expression of multidrug resistance genes, including MDR1 (P-gp). In addition, metformin decreases HIF-1α activation and stability, thereby attenuating hypoxia-driven adaptive responses, such as anaerobic glycolysis and angiogenesis [113].

Metformin inhibits the stimulation-induced activation of the NF-κB, STAT3, and Akt signaling pathways in B-cell chronic lymphocytic leukemia cells [67].

Metformin successfully counteracts and delays the development of acquired resistance to EGFR-tyrosine kinase inhibitors (EGFR-TKIs) in EGFR-mutant lung cancer cells [116]. This reversal of chemoresistance is mechanistically driven by the activation of AMPK, which subsequently suppresses downstream ERK/NF-κB signaling pathways [116].

Given the antagonistic effects associated with NF-κB activation, pharmacological inhibition of this survival pathway remains an important strategy for improving anticancer efficacy. Recent evidence suggests that caffeine can modulate NF-κB signaling and thereby enhance therapeutic responses [117,118]. In breast cancer models, caffeine combined with paclitaxel suppresses NF-κB-dependent prosurvival signaling, restores chemosensitivity, promotes apoptosis, and attenuates pro-inflammatory responses through downregulation of NF-κB expression [117]. Similarly, in colorectal cancer, caffeine has been shown to interfere with the NF-κB signaling network, thereby contributing to the inhibition of tumor progression [118].

The available evidence indicates that itraconazole exerts anticancer activity by inhibiting Hedgehog and indirectly suppressing NF-κB signaling, thereby promoting apoptosis, suppressing cancer cell proliferation, attenuating inflammatory responses, and inducing cell cycle arrest [119].

Nitroglycerin exerts tumor-suppressive effects, at least in part, through NO-dependent modulation of TNFα/TNF-R1 signaling, shifting the canonical pro-survival NF-κB pathway toward a pro-apoptotic pathway and thereby promoting cancer cell death [120]. Nitroglycerin may enhance anticancer therapy by targeting HIF-1α, thereby attenuating hypoxia-driven pro-survival signaling associated with treatment resistance. Furthermore, it may improve the tumor-selective delivery and therapeutic efficacy of anticancer agents while reducing damage to normal tissues [121].

The antitumor activity of disulfiram in gastric cancer has been associated with inhibition of the NF-κB and Wnt/β-catenin signaling pathways. Disulfiram suppresses cancer cell proliferation, migration, and invasion, accompanied by reduced expression of PCNA, Wnt, β-catenin, and NF-κB, thereby promoting apoptosis and limiting tumor progression [122].

Diclofenac inhibits cell proliferation by inducing cell cycle arrest and apoptosis in various cell types, including glioblastoma cells, osteoblasts, vascular smooth muscle cells, human lymphatic endothelial cells, and ovarian cells. These effects are associated with upregulation of the cell cycle inhibitors p21 and p27, downregulation of cyclin D1, and activation of multiple pro-apoptotic mechanisms, including ROS-mediated inhibition of the PI3K/Akt pathway, suppression of NF-κB signaling, modulation of COX activity, proteasome impairment, and activation of the JNK pathway [123].

Evidence indicates that microtubule depolymerization represents an important trigger of NF-κB activation. Accordingly, mebendazole-induced microtubule disruption activates NF-κB in neuroblast cell cultures and in mouse and rat models [124,125], while similar effects have also been reported in HeLa S3 cells following treatment with mebendazole, other benzimidazoles, or cold-induced microtubule depolymerization [126].

Our analyses suggest that disulfiram was the most frequently included agent in metformin-based combinations showing the strongest anticancer activity against hamster fibrosarcoma, as indicated by the lowest *p*-values across tumor endpoints (Table 8 and Table 9). It also consistently appeared in the most effective regimens identified in pairwise comparisons with other active treatments (Table 10).

Metformin represents a cornerstone in drug repurposing strategies in oncology, primarily through inhibition of mitochondrial complex I, resulting in decreased ATP production and subsequent modulation of the AMPK, mTOR and NF-κB signaling axis. Cancer cells frequently exhibit increased reliance on glycolytic metabolism; therefore, the combination of metformin with other repurposed agents may induce a metabolic double blockade, effectively depriving tumor cells of sufficient energy supply, as demonstrated in Table 1 and Table A1 [2,6,7,8,9,10,11,12,13,14,15,16,17,18,19,20,21,22,23,24,25,26,27,28,29,30,31,32,33,34,35,36,37,38,39,40,41,42,43,44,45,46,47,48,49,50,51,52,53,54,55,56,57,58,59,60,61,62,63,64,65,66,67,68,69,70,71,72,73,74,75,76,77,78,79,80,81,82,83,84,85,86,87,88,89,90,91,92,93,94,95,96,97,98,99,100,101,102,103,104,105,106,107,108,109,110,111,112] (Appendix A).

AMPK and Akt function as central but functionally opposing master regulators of cellular metabolism and survival. AMPK serves as an intracellular energy sensor that promotes catabolic processes and energy conservation under conditions of metabolic stress. It negatively regulates mTOR signaling (AMPK\mTOR) and is integrated into multiple downstream signaling networks involving mTOR/NF-κB. In contrast, Akt acts as a pro-growth kinase that inhibits AMPK signaling (Akt\AMPK) and activates mTOR/NF-κB signaling (Akt/mTOR/NF-κB), thereby promoting anabolic processes and cell survival.

The interconnected regulatory network involving AMPK, Akt, mTOR, and NF-κB is summarized in Appendix B based on the literature review [127].

The pronounced preclinical superiority of the metformin and disulfiram combination observed across our evaluated parameters can be attributed to a multi-targeted, potentially synergistic collapse of cancer cell bioenergetics and redox homeostasis. Mechanistically, while metformin selectively inhibits mitochondrial Complex I and suppresses oxidative phosphorylation, it inadvertently forces tumor cells to rely on compensatory glycolysis for survival [128]. Disulfiram effectively counteracts this survival mechanism by inhibiting aldehyde dehydrogenase (ALDH) and key glycolytic enzymes, thereby inducing a devastating dual energetic blockade that starves the tumor cells of ATP [129]. Furthermore, the localized generation of mitochondrial reactive oxygen species (mROS) provoked by metformin is drastically amplified by the pro-oxidant actions of disulfiram [128,129]. This combined assault overwhelms the intracellular antioxidant machinery, driving the cells toward irreversible oxidative stress and apoptosis [129]. Finally, given that disulfiram is a potent inhibitor of ALDH-positive cancer stem cells (CSCs) and both agents downregulate the NF-κB signaling cascade, this combination concurrently dismantles tumor stemness and downstream survival pathways, providing a robust molecular rationale for its exceptional efficacy in the hamster fibrosarcoma model [128,129].

The remarkable potency of the metformin and diclofenac combination against hamster fibrosarcoma observed in this study may stem from a multi-targeted, potentially synergistic mechanism that simultaneously disrupts tumor metabolism and survival signaling. Metformin primarily exerts its antineoplastic effects by inhibiting Complex I of the mitochondrial electron transport chain, thereby impairing oxidative phosphorylation and decreasing ATP production [128]. To survive this energetic stress, tumor cells typically upregulate glycolysis as a metabolic rescue pathway. However, diclofenac has been shown to critically restrict glycolysis and blunt lactate efflux in cancer cells [130]. Diclofenac exhibits antineoplastic potential by inhibiting the COX-2/PGE2 pathway, inducing apoptosis, and altering tumor metabolism, thereby suppressing angiogenesis and reducing immunosuppression in various cancer types [131]. Consequently, it can be presumed that the concurrent administration of both agents potentially induces a severe energy crisis, thereby disrupting the metabolic plasticity of fibrosarcoma cells. Beyond metabolic targeting, this specific drug combination potentially converges on the nuclear factor-kappa B (NF-κB) pathway. The inhibition of NF-κB phosphorylation and nuclear translocation by diclofenac, combined with metformin-mediated suppression of upstream inflammatory signals, offers a strong mechanistic basis for the joint downregulation of essential survival and angiogenic factors in the hamster fibrosarcoma.

In conclusion, our integrated analysis highlights disulfiram and diclofenac as the most promising adjuvants within the top-performing metformin combinations, consistently yielding the highest statistical significance across all evaluated parameters (Table 7, Table 8, Table 9 and Table 10). Based on comparative therapeutic outcomes, the overall statistical ranking of the co-administered agents follows a descending order: disulfiram, diclofenac, nitroglycerin, itraconazole, and caffeine.

Collectively, our data establish a solid rationale for the continued preclinical evaluation of the metformin regimens detailed in Table 7, Table 8, Table 9 and Table 10, serving as a stepping stone toward future well-designed clinical studies that will be essential to define their therapeutic role in fibrosarcoma and other malignancies.

## Figures and Tables

**Table 1:** Anticancer effects of metformin combinations across different cancer types: evidence from in vitro and in vivo studies.

Cancer Type	Metformin in Combination with:	In Vitro (Cell Lines) *	In Vivo	Anticancer Effects, Mechanism and Targeted Signaling Pathways	First Author, Year, Ref. No.
Breast cancer	doxorubicin	MCF-10A, MCF-7, SKBR3, MDA-MB-486	mouse xenograft model (human breast tumors)	selective targeting of cancer stem cells (CSCs)	Hirsch et al. 2009, [9]
Breast cancer	melatonin	—	HER2/ neu transgenic mice	anti-proliferative metabolic regulation; lifespan extension; tumor growth inhibition	Anisimov et al. 2010, [10]
Breast cancer	trastuzumab (TZM)	—	JIMT-1 (HER2+ human breast cancer resistant to TZM) xenograft mice	elimination of CD44^+^CD24^−/low^ CSCs; HER2 signaling suppression	Cufi et al. 2012, [12]
Breast cancer	rapamycin	MCF-7	—	inhibition of cell growth, proliferation, and aromatase; reduction in androgen-to-E2 conversion; sensitization to rapamycin therapy	Rice et al. 2015, [14]
Breast cancer	everolimus	HCC1428, MDA-MB-468, BT549	mice	cancer growth inhibition	Wang Y. et al. 2014, [15]
Breast cancer	doxorubicin	MCF7/ADR	nude mice	tumor growth suppression; P-glycoprotein inhibition; doxorubicin resistance reversal	Li et al. 2018, [21]
Breast cancer (triple-negative breast cancer—TNBC)	cisplatin	Hs 578T, MDA-MB-231, 4T1	mice	cell viability and metastatic effect decrease; RAD51 suppression; sensitization to cisplatin therapy	Lee et al. 2019, [24]
Breast cancer (triple-negative breast cancer—TNBC)	histone deacetylase (HDAC) inhibitors (SAHA)	MDA-MB-231, BT-549, HCC1806, Hs 578T	nude mice	FGFR4 pathway inhibition; tumor growth inhibition	Gu et al. 2025, [25]
Breast cancer, vaginal cancer, colorectal adenocarcinoma	methotrexate	MCF-7, MDA-MB-231, Hela, HT-29	—	cancer cell viability and proliferation decrease; sensitization to methotrexate therapy	Rizvi 2025, [26]
Colon cancer	sirolimus	HT29, SW620, HCT116	nude mice	cell viability inhibition; tumor growth inhibition; mTOR inhibition	Mussin et al. 2017, [30]
Colorectal cancer	niclosamide	DLD-1 (ATCC CCL-221), HEK293, SW480, APC-MIN	mice; patient biopsy samples	Wnt and YAP inhibition; AMPK activation	Kang et al. 2021, [36]
Esophageal cancer	5-fluorouracil	FLO-1, BE3, SKGT-4, OE33, JHESO, OACP, YES-6, KATO-TN	xenograft mice	CSC targeting; PI3K/mTOR signaling pathway inhibition; chemosensitization	Honjo et al. 2014, [42]
Fibrosarcoma	caffeine	BHK-21/C13	Syrian golden hamsters	inhibition of fibrosarcoma growth	Popović D.J. et al. 2018, [46]
Fibrosarcoma	itraconazole	BHK-21/C13; MRC-5, BHK-21/C13, HeLa, HT-29 A549	Syrian golden hamsters	inhibition of fibrosarcoma growth	Popović K.J. et al. 2019, [47]
Fibrosarcoma	nitroglycerin	BHK-21/C13	Syrian golden hamsters	inhibition of fibrosarcoma growth, HIF-1α and downstream targets (VEGF, P-pg-MDRP, MRPs)	Popović K.J. et al. 2020, [48]
Fibrosarcoma	disulfiram	BHK-21/C13	Syrian golden hamsters	inhibition of fibrosarcoma growth, NF-κB, HIF-1α, and downstream targets (VEGF, P-gp, and MRPs)	Popović K.J. et al. 2021, [49]
Fibrosarcoma	diclofenac; caffeine; itraconazole; nitroglycerin; disulfiram; deoxycholic acid	BHK-21/C13	Syrian golden hamsters	reduction in mutational status, tumor proliferation, neoangiogenesis, and glucose/NO metabolism; modulation of apoptosis in correlation with tumor size; inhibition of fibrosarcoma growth	Popovıć J.K. et al. 2024, [50]
Fibrosarcoma	caffeine; itraconazole; nitroglycerin	BHK-21/C13	Syrian golden hamsters	inhibition of fibrosarcoma growth	Popovıć D.J. et al. 2024, [51]
Fibrosarcoma	disulfiram; deoxycholic acid	BHK-21/C13	Syrian golden hamsters	inhibition of fibrosarcoma growth and MAPK/PI3K/AKT/mTOR/NF-κB signaling pathways	Popovıć K.J. et al. 2024, [52]
Glioblastoma	temozolomide and/or irradiation	U87 (ATCC HTB-14), LN18 (ATCC CRL-2610), U251, SF767	nude mice	inhibition of cancer cell growth; reduction in mitochondrial-dependent ATP production and proliferation; induction of cell cycle arrest, autophagy, apoptosis, and cell death; sensitization to temozolomide and/or irradiation therapy	Sesen et al. 2015, [55]
Hepatocellular carcinoma	radiation	Huh7, HepG2, Hep3B	—	radiosensitization; AMPK activation; DNA damage enhancement	Kim et al. 2014, [60]
Hepatocellular carcinoma	sorafenib	MHCC97H	nude mice	tumor suppressor TIP30 increase; proliferation inhibition; lung metastasis reduction	Guo et al. 2016, [61]
Leukemia (chronic lymphocytic leukemia, CLL)	ritonavir	CLL cells from HIV patients	HIV patients	sensitization to ritonavir therapy	Adekola et al. 2015, [66]
Leukemia (chronic lymphocytic leukemia)	fludarabine; ABT-737	cells from the peripheral blood of CLL patients	patients’ peripheral blood	induction of apoptosis; cell cycle inhibition; Mcl-1 downregulation	Bruno et al. 2015, [67]
Lung cancer	doxorubicin	A549, NIH-3		apoptosis increase; tumor growth inhibition; sensitization to doxorubicin therapy	Nazemiyeh et al. 2025, [71]
Lung cancer: non-small-cell lung cancer (NSCLC)	epigallocatechin-3-gallate (EGCG)	A549, H1299, H460, BEAS-2B	nude mice	sensitization to epigallocatechin-3-gallate (EGCG) treatment; Nrf2/HO-1 signaling pathway suppression; ROS increase; tumor growth inhibition	Yu et al. 2017, [74]
Lung cancer: small-cell lung cancer (SCLC)	cisplatin	H446, H526, H446/DDP, H526/DDP	nude mice	suppression of cancer cell growth, EGFR/AKT and mTOR signaling; induction of autophagy, apoptosis, cell cycle arrest, and AMPK; sensitization to cisplatin therapy and alleviation of its toxicity	Xia et al. 2025, [76]
Multiple myeloma, breast, melanoma, and ovarian cancer	ritonavir	KMS11, L363, JJN3, NCI-60	patient samples, mice	Mcl-1 (Bcl-2 family member), AKT, AMPK and mTORC1 suppression; apoptosis induction	Dalva-Aydemir et al. 2015, [78]
Nasopharyngeal carcinoma	radiation	CNE-2, HONE-1, SUNE-1	—	inhibition of DNA repair pathways; radiosensitization	Li et al. 2014, [79]
Ovarian cancer	cisplatin	SKOV3, A2780	Nude mouse xenografts	CSC targeting; AMPK activation; proliferation inhibition	Shank et al. 2012, [84]
Ovarian cancer	paclitaxel	Ovcar-5, Kras/PTEN, SKOV3ip1, Kuramochi, HeyA8, IOSE	mice	cell cycle arrest; sensitization to paclitaxel therapy; tumor reduction	Lengyel et al. 2015, [85]
Ovarian cancer	cisplatin	SKOV3, A2780	mice	inhibition of cancer cell growth, tumor growth, and cancer stem cell (CSC) self-renewal; sensitization to cisplatin therapy	Zhang et al. 2015, [86]
Ovarian cancer	sirtuin 3 (SIRT3)	SKOV3, A2780, HO8910	—	inhibition of cancer cell growth; energy stress and apoptosis induction; AMPK activation	Wu et al. 2018, [87]
Ovarian cancer	fasting cycles	SKOV3, A2780	nude mice	suppression of cancer cell viability and proliferation under low-glucose conditions; induction of apoptosis and ferroptosis	Wu et al. 2025, [88]
Prostate cancer	2-deoxyglucose	LNCaP, P69, PC3, DU145	—	inhibition of glycolysis; induction of metabolic stress and p53-dependent apoptosis	Ben Sahra et al. 2010, [98]
Prostate cancer	enzalutamide	LNCaP, MR49F, C4–2, C4–2R, 22Rv1, LuCaP35CR	nude mice	suppression of cancer cell proliferation and viability; inhibition of mitogen-activated protein kinase (MAPK) signaling; overcoming of enzalutamide resistance	Simpson et al. 2025, [103]
Salivary adenocarcinoma	PP242 (an mTOR inhibitor)	HSY, HSG	nude mice	suppression of cell growth, proliferation, and MYC expression; induction of cell cycle arrest and apoptosis; restoration of P53; enhancement of tumor necrosis	Guo et al. 2015, [107]
Thyroid cancer	2-deoxy-D-glucose	FTC133, BCPAP	patients’ tissue samples	inhibition of cell proliferation; induction of endoplasmic reticulum (ER) stress, autophagy, and cell death	Bikas et al. 2015, [108]
Thyroid carcinoma	chemotherapeutic agent (doxorubicin; cisplatin)	HTh74 HTh74Rdox, SW1736, C643, FTC133, FRTL-5	—	activation of AMPK; induction of apoptosis; inhibition of cancer stem cell (CSC) self-renewal	Chen et al. 2012, [111]

* Note: To ensure absolute fidelity to the primary literature and facilitate accurate cross-referencing, all cell line designations, gene, and protein nomenclatures have been retained exactly as reported in the original cited source publications.

**Table 2:** Experiments conducted to evaluate anticancer effects of metformin (Met) alone and in dual combinations with caffeine (Caf), itraconazole (Itra), nitroglycerin (Nit), disulfiram (Dis), or diclofenac (Dic) on hamster fibrosarcoma at human-equivalent doses. Metformin adjuvant monotherapies and mebendazole (Meb) alone (used in rescue experiments) are also presented.

Experiment No.	Daily Dose (mg/kg)		Animal Weight at Start (g)	Animal Weight at End (g)	No. of Animals per Group	Sex M/F *	Pre- Treatment + Treatment Duration (Days)	Ref. No.
	Met	Other Drug						
1	500	-	97.5 ± 6.12	102 ± 9.5	7	M/F	7 + 14	[45]
2	500	-	84.63 ± 13.04	89.37 ± 15.15	8	M/F	3 + 14	[45]
3	500	-	93.33 ± 17.27	97.17 ± 18.15	6	M/F	0 + 14	[45]
4	500	Caf 100	90.67 ± 11.31	88.00 ± 31.11	6	M/F	3 + 14	[46]
5	500	Itra 250	101.8 ± 12.1	104.3 ± 12.9	6	M/F	3 + 14	[47]
6	250	Itra 250	95.0 ± 6.3	99.5 ± 7.7	6	M/F	3 + 21	[47]
7	500	-	107.3 ± 26.2	118.7 ± 29.5	6	M/F	3 + 14	[47]
8	250	-	85.8 ± 13.5	91.3 ± 8.7	6	M/F	3 + 21	[47]
9	500	Nit 25	61.7 ± 8.1	87.7 ± 6.5	8	M/F	0 + 14	[48]
10	500	Nit 25	61.38 ± 3.42	82.38 ± 3.54	8	M/F	0 + 14	[48]
11	500	-	56.9 ± 4.1	81.5 ± 6.2	8	M/F	0 + 14	[48]
12	500	Nit 25	61.25 ± 5.99	85.63 ± 6.21	8	M/F	0 + 14	[48]
13	1000	-	57.63 ± 5.32	80.63 ± 6.32	8	M/F	0 + 14	[48]
14	500	-	70.6 ± 3.11	89.1 ± 26.9	8	M/F	0 + 19	[49]
15	500	Dis 50	69.8 ± 4.23	94.1 ± 3.94	8	M/F	0 + 19	[49]
16	1000	-	66.3 ± 3.73	89.1 ± 4.12	8	M/F	0 + 19	[49]
17	500	Dic 60	77.0 ± 5.1	94.0 ± 6.6	6	M	0 + 19	[50]
18	-	Caf 100	89.83 ± 13.95	98.17 ± 13.04	6	M/F	3 + 14	[46]
19	-	Itra 250	110.2 ± 34.7	106.0 ± 20.3	6	M/F	3 + 14	[47]
20	-	Itra 250	102.8 ± 20.8	100.3 ± 8.7	6	M/F	3 + 21	[47]
21	-	Nit 25	62.5 ± 2.5	80.0 ± 6.5	8	M/F	0 + 14	[48]
22	-	Nit 50	61.25 ± 3.11	82.63 ± 3.85	8	M/F	0 + 14	[48]
23	-	Dis 50	65.3 ± 4.03	89.4 ± 7.01	8	M/F	0 + 19	[49]
24	-	Dis 100	67.3 ± 4.53	91.3 ± 4.92	8	M/F	0 + 19	[49]
25	-	Dic 60	76.9 ± 4.95	92.2 ± 5.69	6	M	0 + 19	[50]
26	-	Meb 460	62.13 ± 7.79	84.75 ± 5.50	8	M/F	0 + 14	[48]
27	-	Meb 460	69.8 ± 2.92	91.6 ± 3.70	8	M/F	0 + 19	[49]
28	500	Nit 25 Meb 460	58.13 ± 5.82	81.13 ± 7.16	8	M/F	0 + 14	[48]
29	500	Dis 50 Meb 460	68.8 ± 4.77	92.9 ± 5.17	8	M/F	0 + 19	[49]

* M—male, F—female.

**Table 3:** Comparative anticancer effects of metformin alone, in dual combinations (with caffeine, itraconazole, nitroglycerin, disulfiram, or diclofenac), comedicated adjuvants alone, and mebendazole at human-equivalent doses on hamster fibrosarcoma, assessed by control-versus-treatment *p* values for tumor weight, length, volume, burden, density and surface.

Experiment No. *	Control-Versus-Treatment *p* Values						
	Tumor Weight	Tumor Length	Tumor Volume	Tumor Burden	Tumor Density	Tumor Surface	Ref. No.
1	0.0272		0.0274				[45]
2	0.1200		0.0739				[45]
3	0.4590		0.4583				[45]
4	0.0490		0.0441				[46]
5	0.009	0.006	0.0095	0.010	0.009	0.0085	[47]
6	0.035	0.045	0.036	0.049	0.005	0.047	[47]
7	0.999	0.311	0.936	0.850	0.025	0.592	[47]
8	0.201	0.133	0.120	0.833	0.480	0.351	[47]
9	0.015	0.0168	0.030	0.025	0.003	0.0039	[48]
10	0.0051	0.0046	0.0056		0.0069		[48]
11	0.650	0.849	0.658	0.927	0.899	0.115	[48]
12	0.0099	0.002	0.029		0.035		[48]
13	0.9297	0.6000	1.0000		0.8030		[48]
14	0.283	0.845	0.404	0.442	0.554	0.699	[49]
15	0.00013	0.00083	0.00055	0.00030	0.01109	0.00048	[49]
16	0.0860	0.00970	0.2610	0.1980	0.6920	0.00865	[49]
17	0.00080	0.0013	0.0009	0.0007	0.0188	0.0009	[50]
18	0.8440		0.8852				[46]
19	0.6100		0.600				[47]
20	0.610		0.551				[47]
21	0.270		0.209				[48]
22	0.6443		0.6680				[48]
23	0.597		0.901				[49]
24	0.287		0.283				[49]
25	0.16483		0.14389				[50]
26	0.375		0.337				[48]
27	0.211		0.411				[49]
28	0.850		0.790				[48]
29	0.922		0.999				[49]

* Experiments numbered 1–29 in Table 2.

**Table 4:** Comparative anticancer effects of metformin alone, in dual combinations (with caffeine, itraconazole, nitroglycerin, disulfiram, or diclofenac), comedicated adjuvants alone, and mebendazole at human-equivalent doses on hamster fibrosarcoma, assessed by control-versus-treatment *p* values for the expression of the tumor immunohistochemical markers Ki-67, PCNA, GLUT1, iNOS, CD34, CD31, COX4, and cytochrome C.

Experiment No. *	Control-Versus-Treatment *p* Values								Ref. No.
	Ki-67	PCNA	GLUT1	iNOS	CD34	CD31	COX4	Cytochrome C	
1	0.000								[45]
2	0.1478								[45]
3	0.1240								[45]
4	0.0199								[46]
5	0.0065		0.041	0.037	0.035		0.034		[47]
6	0.044		0.044	0.040	0.039		0.042		[47]
7	0.250		0.910	0.930	0.770		0.980		[47]
8	0.479		0.510	0.980	0.870		0.980		[47]
9	0.0031	0.0081	0.0089	0.0082	0.0150	0.0155	0.0087	0.0139	[48]
10	0.0044								[48]
11	0.8970	0.9690	0.4500	0.4201	0.5980	0.4020	0.1116	0.9990	[48]
12	0.003								[48]
13	0.8787								[48]
14	0.90862	0.90859	0.50405	0.41005	0.54100	0.55200	0.55011	0.75341	[49]
15	0.00011	0.00077	0.00150	0.000179	0.00514	0.00091	0.00103	0.00099	[49]
16	0.80910	0.99605	0.32900	0.39202	0.20710	0.48907	0.58615	0.80700	[49]
17	0.0008	0.00090	0.0002	0.0011	0.0003	0.0012	0.0007	0.00050	[50]
18	0.3290								[46]
19	0.170								[47]
20	0.535								[47]
21	0.680								[48]
22	0.7515								[48]
23	0.58301								[49]
24	0.79852								[49]
25	0.4600								[50]
26	0.690								[48]
27	0.44127								[49]
28	0.800								[48]
29	0.41400								[49]

* Experiments numbered 1–29 in Table 2.

**Table 5:** Comparative anticancer effects of metformin alone, in dual combinations (with caffeine, itraconazole, nitroglycerin, disulfiram, or diclofenac), comedicated adjuvants alone, and mebendazole at human-equivalent doses on hamster fibrosarcoma, presented as percentages of the corresponding control mean ± SD for tumor weight, length, volume, burden, density and surface.

Experiment No. *	Percent of the Corresponding Control Mean ± SD						Ref. No.
	Tumor Weight	Tumor Length	Tumor Volume	Tumor Burden	Tumor Density	Tumor Surface	
1	12.29 ± 14.11		12.27 ± 14.13				[45]
2	43.24 ± 30.19		33.48 ± 30.17				[45]
3	69.03 ± 38.89		68.93 ± 38.86				[45]
4	16.47 ± 12.49		14.16 ± 10.74				[46]
5	16.20 ± 7.44	50.00 ± 12.85	16.77 ± 7.78	13.89 ± 8.33	94.47 ± 1.84	10.83 ± 4.17	[47]
6	24.79 ± 8.67	63.52 ± 14.66	25.78 ± 8.82	35.71 ± 8.62	95.41 ± 2.11	24.14 ± 7.00	[47]
7	100.33 ± 68.48	88.33 ± 16.25	93.41 ± 68.86	83.33 ±16.25	96.33 ± 2.48	83.33 ± 41.66	[47]
8	57.62 ± 26.85	80.78 ± 14.33	57.39 ± 25.51	90.91 ±49.09	98.16 ± 3.21	59.65 ± 21.05	[47]
9	53.78 ± 28.09	71.65 ± 22.44	57.85 ± 29.75	56.67 ± 31.67	94.95 ± 20.18	39.20 ± 20.20	[48]
10	20.68 ± 14.00	45.70 ± 22.27	23.71 ± 16.12		86.64 ± 4.64		[48]
11	90.64 ± 36.70	97.64 ± 16.93	90.50 ± 36.36	95.00 ± 36.67	101.83 ± 32.11	61.60 ± 32.02	[48]
12	65.37 ± 19.67	73.95 ±13.50	69.33 ± 16.33		91.31 ± 7.38		[48]
13	101.11 ± 24.93	96.14 ± 15.76	100.00 ± 28.00		100.90 ± 6.97		[48]
14	91.55 ± 15.75	99.08 ± 13.85	91.90 ± 16.48	91.81 ± 14.28	98.37 ± 4.86	94.60 ± 25.22	[49]
15	14.84 ± 6.44	39.69 ± 12.92	16.76 ± 7.54	13.26 ± 5.71	89.34 ± 8.91	9.01 ± 4.50	[49]
16	88.81 ± 12.78	77.23 ± 17.23	88.27 ± 15.36	90.00 ± 17.14	96.36 ± 6.48	68.02 ± 24.32	[49]
17	6.98 ± 8.90	40.23 ± 18.12	6.69 ± 9.86	6.28 ± 8.99	91.05 ± 1.88	14.99 ± 5.66	[50]
18	91.41 ± 51.58		106.56 ± 59.45				[46]
19	80.74 ± 55.55		81.44 ± 56.89				[47]
20	82.54 ± 12.27		84.40 ± 13.43				[47]
21	73.78 ± 38.20		72.73 ± 37.19				[48]
22	94.18 ± 27.93		93.67 ± 26.67				[48]
23	96.35 ± 22.60		98.04 ± 24.02				[49]
24	91.32 ± 14.61		90.50 ± 14.25				[49]
25	78.95 ± 26.32		75.76 ± 25.15				[50]
26	79.93 ± 38.31		78.57 ± 38.90				[48]
27	89.04 ± 21.23		90.78 ± 22.35				[49]
28	97.63 ± 38.99		93.88 ± 38.78				[48]
29	100.68 ± 6.39		100.00 ± 8.66				[49]

* Experiments numbered 1–29 in Table 2.

**Table 6:** Comparative anticancer effects of metformin alone, in dual combinations (with caffeine, itraconazole, nitroglycerin, disulfiram, or diclofenac), comedicated adjuvants alone, and mebendazole at human-equivalent doses on hamster fibrosarcoma, presented as percentages of the corresponding control mean ± SD for the expression of the tumor immunohistochemical markers Ki-67, PCNA, GLUT1, iNOS, CD34, CD31, COX4, and cytochrome C.

Exp. No. *	Tumor Immunohistochemical Marker Expression Percent of the Corresponding Control Mean ± SD								Ref. No.
	Ki-67	PCNA	GLUT1	iNOS	CD34	CD31	COX4	Cytochrome C	
1	45.57 ± 15.89								[45]
2	77.73 ± 32.05								[45]
3	77.46 ± 28.54								[45]
4	72.05 ± 19.06								[46]
5	44.4 ± 22.22		26.92 ± 19.23	26.31 ± 13.16	17.39 ± 10.87		26.32 ± 18.42		[47]
6	55.00 ± 30.00		32.14 ± 13.57	35.14 ± 16.04	28.00 ± 10.00		31.76 ± 10.73		[47]
7	83.33 ± 36.11		88.46 ± 26.92	89.47 ± 42.11	113.61 ± 50.00		105.26 ± 42.11		[47]
8	87.50 ± 16.00		67.86 ± 32.14	107.41 ± 37.04	92.31 ± 42.31		100.00 ± 29.79		[47]
9	45.45 ± 39.55	35.45 ± 31.82	45.83 ± 12.86	35.00 ± 17.50	39.33 ± 25.00	40.74 ± 32.73	25.50 ± 25.00	26.98 ± 26.59	[48]
10	32.87 ± 13.47								[48]
11	95.55 ± 41.90	94.74 ± 36.84	85.71 ± 32.65	85.00 ± 41.60	92.83 ± 28.29	82.16 ± 42.43	92.86 ± 47.62	97.71 ± 63.41	[48]
12	52.66 ± 19.59								[48]
13	97.42 ± 36.19								[48]
14	100.63 ± 17.81	101.61 ± 29.81	110.71 ± 39.28	111.54 ± 32.31	110.77 ± 43.08	106.02 ± 29.64	105.97 ± 29.25	103.64 ± 22.54	[49]
15	15.78 ± 15.62	16.45 ± 13.55	20.71 ± 10.71	21.15 ± 13.08	13.85 ± 7.38	0.24 ± 0.48	13.43 ± 0.83	10.54 ± 0.22	[49]
16	96.87 ± 21.56	100.97 ± 18.39	113.57 ± 25.00	107.69 ± 19.23	123.08 ± 22.15	105.78 ± 24.34	105.73 ± 20.89	102.91 ± 10.54	[49]
17	32.48 ± 10.02	31.23 ± 10.10	36.00 ± 15.20	35.90 ± 11.20	28.90 ± 10.50	31.07 ± 10.27	22.99 ± 11.08	23.86 ± 17.02	[50]
18	87.40 ± 25.24								[46]
19	78.38 ± 32.43								[47]
20	90.0 ± 12.50								[47]
21	83.33 ± 41.67								[48]
22	94.38 ± 39.69								[48]
23	95.24 ± 22.62								[49]
24	101.59 ± 13.57								[49]
25	85.0 ± 33.90								[50]
26	90.97 ± 38.91								[48]
27	92.06 ± 29.33								[49]
28	94.84 ± 37.31								[48]
29	101.56 ± 4.13								[49]

* Experiments numbered 1–29 in Table 2.

**Table 7:** The 95% confidence intervals (95% CI) and interval widths for the primary endpoints (tumor weight and tumor volume) and the secondary endpoint (Ki-67 expression).

Exp. No. *	Tumor Weight		Tumor Volume		Ki-67		Ref. No.
	95% CI	CI Width	95% CI	CI Width	95% CI	CI Width	
1	[1.3835, 23.1965]	21.81	[1.3671, 23.1729]	21.81	[22.6133, 68.5267]	45.91	[45]
2	[−11.2698, 97.7498]	109.02	[−3.2365, 70.1965]	73.43	[−27.5321, 182.9921]	210.52	[45]
3	[−113.6842, 251.7442]	365.43	[−113.2355, 251.0955]	364.33	[−21.2409, 176.1609]	197.40	[45]
4	[0.0719, 32.8681]	32.80	[0.3738, 27.9462]	27.57	[11.3953, 132.7047]	121.31	[46]
5	[4.0441, 28.3559]	24.31	[4.0964, 29.4436]	25.35	[12.4228, 76.3772]	63.95	[47]
6	[1.7444, 47.8356]	46.09	[1.6834, 49.8766]	48.19	[1.4771, 108.5229]	107.05	[47]
7	[−156802.22, 157002.88]	313805.10	[−2186.64, 2373.46]	4560.10	[−58.5798, 225.0398]	283.62	[47]
8	[−30.6998, 145.9398]	176.64	[−21.0540, 182.6140]	203.67	[−154.7619, 329.7619]	484.52	[47]
9	[10.4443, 97.1157]	86.67	[5.6005, 110.0995]	104.50	[15.3309, 75.5691]	60.24	[48]
10	[6.2074, 35.1526]	28.95	[6.9353, 40.4847]	33.55	[10.2486, 55.4914]	45.24	[48]
11	[−300.8743, 482.1543]	783.03	[−310.1992, 491.1992]	801.40	[−1351.1493, 1542.2493]	2893.40	[48]
12	[15.6957, 115.0443]	99.35	[7.0961, 131.5639]	124.47	[17.8816, 87.4384]	69.56	[48]
13	[−2145.2152, 2347.4300]	4492.65	[−1563779, 1563979]	3127758.00	[−1153.70, 1348.5400]	2502.24	[48]
14	[−75.5860, 258.6860]	334.27	[−123.9470, 307.7470]	431.69	[−1617.75, 1819.01]	3436.76	[49]
15	[7.2388, 22.4412]	15.20	[7.2527, 26.2673]	19.01	[7.2829, 23.7771]	16.49	[49]
16	[−12.5757, 190.1957]	202.77	[−65.6485, 242.1885]	307.84	[−689.0369, 882.7769]	1571.81	[49]
17	[2.8996, 11.0604]	8.16	[2.7406, 10.6394]	7.90	[13.4928, 51.4672]	37.97	[50]
18	[−819.0682, 1001.8882]	1820.96	[−1340.018, 1553.138]	2893.16	[−88.093, 262.893]	350.99	[46]
19	[−229.5102, 390.9902]	620.50	[−222.9503, 385.8303]	608.78	[−33.5748, 190.3348]	223.91	[47]
20	[−234.6269, 399.7069]	634.33	[−193.0350, 361.8350]	554.87	[−194.3366, 374.3366]	568.67	[47]
21	[−57.3175, 204.8775]	262.20	[−40.7369, 186.1969]	226.93	[−312.6490, 479.3090]	791.96	[48]
22	[−305.6353, 493.9953]	799.63	[−334.3913, 521.7313]	856.12	[−489.7906, 678.5506]	1168.34	[48]
23	[−260.8261, 453.5261]	714.35	[−1446.6653, 1642.7553]	3089.42	[−244.7831, 435.2631]	680.05	[49]
24	[−76.7863, 259.4263]	336.21	[−74.7191, 255.7191]	330.44	[−678.4538, 881.6338]	1560.09	[49]
25	[−32.4546, 190.3546]	222.81	[−25.8438, 177.3638]	203.21	[−140.4866, 310.4866]	450.97	[50]
26	[−96.6618, 256.5218]	353.18	[−81.8254, 238.9654]	320.79	[−356.0624, 538.0024]	894.06	[48]
27	[−50.4831, 228.5631]	279.05	[−125.6429, 307.2029]	432.85	[−142.2593, 326.3793]	468.64	[49]
28	[−914.1950, 1109.4550]	2023.65	[−597.0601, 784.8201]	1381.88	[−638.8818, 828.5618]	1467.44	[48]
29	[−1914.6765, 2116.0365]	4030.71	[−156286.47, 156486.47]	312772.94	[−142.1220, 345.2420]	487.36	[49]

* Experiments numbered 1–29 in Table 2.

**Table 8:** Ranking of statistically significant (*p* < 0.05) anticancer treatments (numbered 1–17 in Table 2) against hamster fibrosarcoma at human-equivalent doses using metformin (Met) alone and in dual combinations with caffeine (Caf), itraconazole (Itra), nitroglycerin (Nit), disulfiram (Dis), or diclofenac (Dic) based on ascending *p* values for tumor endpoints: weight, length, volume, burden, density, and surface area.

Rank *	Tumor Weight	Tumor Length	Tumor Volume	Tumor Burden	Tumor Density	Tumor Surface
1	(15) Met 500 + Dis 50 0.00013	(15) Met 500 + Dis 50 0.00083	(15) Met 500 + Dis 50 0.00055	(15) Met 500 + Dis 50 0.0003	(9) Met 500 + Nit 25 0.003	(15) Met 500 + Dis 50 0.00048
2	(17) Met 500 + Dic 60 0.00080	(17) Met 500 + Dic 60 0.0013	(17) Met 500 + Dic 60 0.0009	(17) Met 500 + Dic 60 0.0007	(6) Met 250 + Itra 250 0.005	(17) Met 500 + Dic 60 0.0009
3	(10) Met 500 + Nit 25 0.0051	(12) Met 500 + Nit 25 0.002	(10) Met 500 + Nit 25 0.0056	(5) Met 500 + Itra 250 0.01	(10) Met 500 + Nit 25 0.0069	(9) Met 500 + Nit 25 0.0039
4	(5) Met 500 + Itra 250 0.009	(10) Met 500 + Nit 25 0.0046	(5) Met 500 + Itra 250 0.0095	(9) Met 500 + Nit 25 0.025	(5) Met 500 + Itra 250 0.009	(5) Met 500 + Itra 250 0.0085
5	(12) Met 500 + Nit 25 0.0099	(5) Met 500 + Itra 250 0.006	(1) Met 500 (7+14 d) 0.0274	(6) Met 250 + Itra 250 0.049	(15) Met 500 + Dis 50 0.01109	(16) Met 1000 (19 d) 0.00865
6	(9) Met 500 + Nit 25 0.015	(16) Met 1000 (19 d) 0.0097	(12) Met 500 + Nit 25 0.029		(17) Met 500 + Dic 60 0.0188	(6) Met 250 + Itra 250 0.047
7	(1) Met 500 (7+14 d) 0.0272	(9) Met 500 + Nit 25 0.0168	(9) Met 500 + Nit 25 0.030		(7) Met 500 (3+14 d) 0.025	
8	(6) Met 250 + Itra 250 0.035	(6) Met 250 + Itra 250 0.045	(6) Met 250 + Itra 250 0.036		(12) Met 500 + Nit 25 0.035	
9	(4) Met 500 + Caf 100 0.0490		(4) Met 500 + Caf 100 0.0441			

* Treatments are ranked in ascending order of their *p* values (Rank 1 indicates the highest statistical significance).

**Table 9:** Ranking of statistically significant (*p* < 0.05) anticancer treatments (numbered 1–17 in Table 2) against hamster fibrosarcoma at human-equivalent doses using metformin (Met) alone and in dual combinations with caffeine (Caf), itraconazole (Itra), nitroglycerin (Nit), disulfiram (Dis), or diclofenac (Dic) based on ascending *p* values for tumor immunohistochemical markers Ki-67, PCNA, GLUT1, iNOS, CD34, CD31, COX4, and cytochrome C.

Rank *	Ki-67	PCNA	GLUT1	iNOS	CD34	CD31	COX4	Cytochrome C
1	(1) Met (7+14 d) 0	(15) Met + Dis 0.00077	(17) Met + Dic 0.0002	(15) Met + Dis 0.000179	(17) Met + Dic 0.0003	(15) Met + Dis 0.00091	(17) Met + Dic 0.0007	(17) Met + Dic 0.0005
2	(15) Met + Dis 0.00011	(17) Met + Dic 0.00090	(15) Met + Dis 0.0015	(17) Met + Dic 0.0011	(15) Met + Dis 0.00514	(17) Met + Dic 0.0012	(15) Met + Dis 0.00103	(15) Met + Dis 0.00099
3	(17) Met + Dic 0.0008	(9) Met + Nit 0.00810	(9) Met + Nit 0.0089	(9) Met + Nit 0.0082	(9) Met + Nit 0.015	(9) Met + Nit 0.0155	(9) Met + Nit 0.0087	(9) Met + Nit 0.0139
4	(12) Met + Nit 0.0030		(5) Met + Itra 0.0410	(5) Met + Itra 0.037	(5) Met + Itra 0.035		(5) Met + Itra 0.034	
5	(9) Met + Nit 0.0031		(6) Met + Itra 0.0440	(6) Met + Itra 0.040	(6) Met + Itra 0.039		(6) Met + Itra 0.042	
6	(10) Met + Nit 0.0044							
7	(5) Met + Itra 0.0065							
8	(4) Met + Caf 0.0199							
9	(6) Met + Itra 0.0440							

* Treatments are ranked in ascending order of their *p* values (Rank 1 indicates the highest statistical significance).

**Table 10:** Significant *p* values (*p* < 0.05) from pairwise comparisons among effective anticancer treatments (Table 8 and Table 9), using tumor endpoint values expressed as percentages of control mean (± SD).

Experiments * (Treatments) Comparison (Superior Efficacy of the First)	Statistically Significant *p*-Values (*p* < 0.05)
**Tumor length**	
(15) Met 500 + Dis 50/(16) Met 1000 (19 d)	0.0002
(15) Met 500 + Dis 50/(12) Met 500 + Nit 25	0.0001
(15) Met 500 + Dis 50/(6) Met 250 + Itra 250	0.0073
(17) Met 500 + Dic 60/(12) Met 500 + Nit 25	0.0017
(17) Met 500 + Dic 60/(6) Met 250 + Itra 250	0.0344
(17) Met 500 + Dic 60/(16) Met 1000 (19 d)	0.0021
**Tumor surface area**	
(15) Met 500 + Dis 50/(17) Met 500 + Dic 60	0.0475
(15) Met 500 + Dis 50/(9) Met 500 + Nit 25	0.0014
(15) Met 500 + Dis 50/(6) Met 250 + Itra 250	0.0003
(15) Met 500 + Dis 50/(16) Met 1000 (19 d)	< 0.0001
(17) Met 500 + Dic 60/(9) Met 500 + Nit 25	0.0152
(17) Met 500 + Dic 60/(6) Met 250 + Itra 250	0.0320
(17) Met 500 + Dic 60/(16) Met 1000 (19 d)	0.0002
(9) Met 500 + Nit 25/(16) Met 1000 (19 d)	0.0218
(6) Met 250 + Itra 250/(16) Met 1000 (19 d)	0.0011
**Ki-67**	
(15) Met 500 + Dis 50/(17) Met 500 + Dic 60	0.0418
(15) Met 500 + Dis 50/(1) Met 500 (7+14 d)	0.0026
(15) Met 500 + Dis 50/(12) Met 500 + Nit 25	0.0010
(15) Met 500 + Dis 50/(4) Met 500 + Caf 100	<0.00001
(15) Met 500 + Dis 50/(6) Met 250 + Itra 250	0.0077
(1) Met 500 (7+14 d)/(4) Met 500 + Caf 100	0.0194
(17) Met 500 + Dic 60/(12) Met 500 + Nit 25	0.0408
(17) Met 500 + Dic 60/(4) Met 500 + Caf 100	0.0011
**PCNA**	
(15) Met 500 + Dis 50/(17) Met 500 + Dic 60	0.0450
**GLUT1**	
(15) Met 500 + Dis 50/(17) Met 500 + Dic 60	0.0467
(15) Met 500 + Dis 50/(9) Met 500 + Nit 25	0.0008
**iNOS**	
(15) Met 500 + Dis 50/(17) Met 500 + Dic 60	0.0469
**CD34**	
(15) Met 500 + Dis 50/(17) Met 500 + Dic 60	0.0082
(15) Met 500 + Dis 50/(9) Met 500 + Nit 25	0.0152
(15) Met 500 + Dis 50/(6) Met 250 + Itra 250	0.0099
**CD31**	
(15) Met 500 + Dis 50/(17) Met 500 + Dic 60	<0.0001
(15) Met 500 + Dis 50/(9) Met 500 + Nit 25	0.0035
**COX4**	
(15) Met 500 + Dis 50/(17) Met 500 + Dic 60	0.0295
(15) Met 500 + Dis 50/(6) Met 250 + Itra 250	0.0004
**Cytochrome C**	
(15) Met 500 + Dis 50/(17) Met 500 + Dic 60	0.0444

* Experiments numbered 1–17 in Table 2.

## Data Availability

No new data were created or analyzed in this study. Data sharing is not applicable to this article.

## Abbreviations

The following abbreviations are used in this manuscript:

**ABC Transporters**	**ATP-Binding Cassette Transporters**
ABCB1	ATP-binding cassette sub-family B member 1 (P-glycoprotein, MDR1)
Akt	Protein kinase B (PKB)
AMPK	AMP-activated protein kinase
ATG	Autophagy-related protein
ATG3	Autophagy-related protein 3
ATG4D	Autophagy-related protein 4D
ATG5	Autophagy-related protein 5
ATG12	Autophagy-related protein 12
ATG16L	Autophagy-related protein 16-like
Bcl-2	B-cell lymphoma 2
Beclin-1	Autophagy-related protein Beclin-1
CD31	Cluster of differentiation 31 (Platelet endothelial cell adhesion molecule-1, PECAM-1)
CDK4	Cyclin-dependent kinase 4
Cyclin B1	G2/mitotic-specific regulatory cyclin B1 protein
Cyclin D1	G1/S-specific regulatory cyclin D1 protein
Cyclins	Cyclin family proteins, regulatory proteins controlling cell cycle progression
EGF	Epidermal growth factor
EGFR	Epidermal growth factor receptor
ERK(1,2)	Extracellular signal-regulated kinase (1 and 2)
FGF	Fibroblast growth factor
HIF	Hypoxia-inducible factor
HIF-1α	Hypoxia-inducible factor 1 alpha
HIF-2α	Hypoxia-inducible factor 2 alpha
IGF	Insulin-like growth factor
IGF-1	Insulin-like growth factor 1
IGF-2	Insulin-like growth factor 2
IKK	IκB kinase
iNOS	Inducible nitric oxide synthase
IκB	Inhibitor of nuclear factor kappa B
IκBα	Inhibitor of nuclear factor kappa B alpha
JAK	Janus kinase
JAK2	Janus kinase 2
JNK	c-Jun N-terminal kinase
Klf4	Kruppel-like factor 4
MAP2	Microtubule-associated protein 2
MAPK	Mitogen-activated protein kinase
MDPRs	Multidrug resistance proteins
MDR	Multidrug resistance
MDR1	Multidrug resistance protein 1 (ABCB1)
MDR1-ABCB1	P-glycoprotein encoded by ABCB1 gene
MEK	Mitogen-activated protein kinase kinase (MAPK/ERK kinase)
MEK1	Mitogen-activated protein kinase kinase 1 (MAPK/ERK kinase 1)
mTOR	Mechanistic target of rapamycin (formerly mammalian target of rapamycin)
MYC	MYC proto-oncogene (Myelocytomatosis oncogene), bHLH transcription factor
NF-κB	Nuclear factor kappa-light-chain-enhancer of activated B cells
NO	Nitric oxide
NRF1	Nuclear respiratory factor 1
Oct4	Octamer-binding transcription factor 4 (POU5F1)
p21	Cyclin-dependent kinase inhibitor 1A (CDKN1A)
p27	Cyclin-dependent kinase inhibitor 1B (CDKN1B)
p38	p38 mitogen-activated protein kinase
p53	Tumor suppressor protein p53
PAK1	P21-activated kinase 1
PCNA	Proliferating cell nuclear antigen
PDGF	Platelet-derived growth factor
P-gp	P-glycoprotein (ABCB1)
PI3K	Phosphoinositide 3-kinase
PKB	Protein kinase B (Akt)
PKC	Protein kinase C
PTEN	Phosphatase and tensin homolog
Raf	Rapidly accelerated fibrosarcoma kinase
Raf1	RAF proto-oncogene serine/threonine-protein kinase
RAS	Rat sarcoma viral oncogene homolog
ROS	Reactive oxygen species
RTKs	Receptor tyrosine kinases
Sox2	SRY-box transcription factor 2
STAT	Signal transducer and activator of transcription
STAT3	Signal transducer and activator of transcription 3
STAT5	Signal transducer and activator of transcription 5
STATs	Signal transducer and activator of transcription proteins
TGF-α	Transforming growth factor-alpha
TGF-β	Transforming growth factor-beta
TLR4	Toll-like receptor 4
TNF	Tumor necrosis factor
VEGF	Vascular endothelial growth factor
VEGFR	Vascular endothelial growth factor receptor
Wnt	Wingless-related integration site
β-catenin	Catenin beta 1 (CTNNB1)

## Appendix A

**Table A1:** Anticancer effects of metformin across different cancer types: evidence from in vitro and in vivo studies.

Cancer Type	Treatment: Metformin Alone and/or Combined with:	In Vitro (Cell Lines) *	In Vivo	Anticancer Effects, Mechanism and Targeted Signaling Pathways	First Author, Year, Ref. No.
Bladder cancer	—	T24, 5637	—	cancer cell proliferation, migration and growth inhibition; apoptosis induction; PI3K/AKT/mTOR pathway inhibition	Shen et al. 2022, [6]
Bladder cancer	—	T24, 5637, MB49	mice	cancer cell growth and proliferation inhibition; PD-L1 decrease	Yeh et al. 2025, [7]
Breast cancer	—	MCF-7, BT-474, SKBR3	—	MAPK, Akt and mTOR inhibition; AMPK activation; dose dependent G1 cell-cycle arrest	Alimova et al. 2009, [8]
Breast cancer	doxorubicin	MCF-10A, MCF-7, SKBR3, MDA-MB-486	Mouse xenograft model (human breast tumors)	selective targeting of cancer stem cells (CSCs)	Hirsch et al. 2009, [9]
Breast cancer	melatonin	—	HER2/neu transgenic mice	anti-proliferative metabolic regulation; lifespan extension; ↓tumor growth	Anisimov et al. 2010, [10]
Breast cancer	—	human breast adipose stromal cells	—	AMPK activation; aromatase suppression	Brown et al. 2010, [11]
Breast cancer	trastuzumab (TZM)	—	JIMT-1 (HER2+ human breast cancer resistant to TZM) xenograft mice	elimination of CD44^+^CD24^−/low^ CSCs; HER2 signaling suppression	Cufi et al. 2012, [12]
Breast cancer	—	BT-474, SKBR3, 78617 cell line derived from MMTV-ErbB2 transgenic mice	mice	ErbB2, EGFR, AKT, mTOR, and STAT3 inhibition; inhibition of CSC self-renewal/proliferation	Zhu et al. 2014, [13]
Breast cancer	rapamycin	MCF-7	—	inhibition of cell growth, proliferation, and aromatase; reduction in androgen-to-E2 conversion; sensitization to rapamycin therapy	Rice et al. 2015, [14]
Breast cancer	everolimus	HCC1428, MDA-MB-468, BT549	mice	cancer growth inhibition	Wang Y. et al. 2014, [15]
Breast cancer	—	MDA-MB-453, 4T1	mice	tumor angiogenesis suppression, HER2/HIF-1alpha/VEGF signaling inhibition	Wang J. et al. 2015, [16]
Breast cancer	—	MDA-MB-231, MDA-MB-468, MCF-7	—	reduction in cancer cell viability; miR-26a increase; Bcl-2, EZH2, and PTEN decrease	Cabello et al. 2016, [17]
Breast cancer	—	MDA-MB-231, NMuMG, MCF10A	type 2 diabetic patients serum samples, mice	AMPK activation; inhibition of TGF-β signaling pathway	Li et al. 2016, [18]
Breast cancer	—	MCF7, HTB-126	—	AMPK activation; mTOR signaling attenuation	Sacco et al. 2016, [19]
Breast cancer	—	MCF-7, MCF-10	—	inhibition of cancer cell proliferation; induction of apoptosis and cell cycle arrest; upregulation of p21, p53, and Bax; downregulation of cyclin D1, Akt, and Bcl-2	Al-Zaidan et al. 2017, [20]
Breast cancer	doxorubicin	MCF7/ADR	nude mice	tumor growth suppression; P-glycoprotein inhibition; doxorubicin resistance reversal	Li et al. 2018, [21]
Breast cancer	—	MCF-7, 4T1	patient tissues; nude mice	inhibition of cancer cell proliferation, cancer growth and COX2; AMPK activation	Shi et al. 2021, [22]
Breast cancer	—	MCF-7	—	inhibition of cancer cell proliferation	Venugopal et al. 2026, [23]
Breast cancer (triple-negative breast cancer—TNBC)	cisplatin	Hs 578T, MDA-MB-231, 4T1	mice	cell viability and metastatic effect decrease; RAD51 suppression; sensitization to cisplatin therapy	Lee et al. 2019, [24]
Breast cancer (triple-negative breast cancer—TNBC)	histone deacetylase (HDAC) inhibitors (SAHA)	MDA-MB-231, BT-549, HCC1806, Hs 578T	nude mice	inhibition of FGFR4 pathway and tumor growth	Gu et al. 2025, [25]
Breast cancer, vaginal cancer, colorectal adenocarcinoma	methotrexate	MCF-7, MDA-MB-231, Hela, HT-29	—	cancer cell viability and proliferation decrease; sensitization to methotrexate therapy	Rizvi 2025, [26]
Cervical cancer	—	SiHa, HeLa	nude mice	suppression of cancer growth, angiogenesis, migration and invasion; disruption of the nuclear MALAT1/miR-142-3p sponge mechanism; induction of HMGA2	Xia et al. 2018, [27]
Cervical cancer (HeLa-derived; HEP-2 cell line)	—	HEP-2 (originated from laryngeal carcinoma and cervical cancer)	—	enhanced cytotoxicity of new nanospanlastic metformin formulation; reduction in live cancer cell percentage; downregulation of Bcl-2, cyclin D1, and mTOR; upregulation of BAX, and caspases-3, -8, and -9	Raafat et al. 2023, [28]
Colon cancer	—	LoVo (CCL-229)	—	inhibition of cancer cell proliferation	He et al. 2015, [29]
Colon cancer	sirolimus	HT29, SW620, HCT116	nude mice	inhibition of cell viability, mTOR and tumor growth	Mussin et al. 2017, [30]
Colon cancer	—	Ht29	—	inhibition of cell growth, NRF-2 and NF-κB; induction of apoptosis and autophagy	Sena et al. 2018, [31]
Colorectal cancer	—	—	Azoxymethane-treated mice	AMPK activation; ↓mTOR; suppression of early carcinogenesis	Hosono et al. 2010, [32]
Colorectal cancer	—	HCT116	mice	AMPK activation	Dowling et al. 2016, [33]
Colorectal cancer	—	HT29, HCT116, HCT116 p53^−/−^ CRC	—	activation of AMPK; induction of cell cycle arrest and ROS production; suppression of cell proliferation, migration, and the mTOR pathway; downregulation of c-Myc and IGF-1R	Mogavero et al. 2017, [34]
Colorectal cancer	—	HT29, DLD-1	nude mice	cancer stem cell (CSC) suppression; AMPK activation; mTOR inhibition; prenylation (mevalonate pathway protein) inhibition	Seo et al. 2020, [35]
Colorectal cancer	niclosamide	DLD-1 (ATCC CCL-221), HEK293, SW480, APC-MIN	mice; patient biopsy samples	Wnt and YAP inhibition; AMPK activation	Kang et al. 2021, [36]
Colorectal carcinoma, lung adenocarcinoma	—	HCT 116 p53^−/−^, HCT116 p53^+/+^, A549, A549, CCL16, CCL16-B2, CCL16-NDI1	mice	mitochondrial complex I (NADH dehydrogenase) and HIF-1 inhibition, cellular proliferation inhibition	Wheaton et al. 2014, [37]
Endometrial adenocarcinoma	—	Ishikawa, KLE, ECC-1	patient tissues	βKlotho (fibroblast growth factors – FGFs coreceptor) increase; ERK1/2 decrease; AMPKα signaling activation	Liu et al. 2016, [38]
Endometrial cancer	—	HEC-1B, Ishikawa	humans (patient tissues)	AMPK activation, MAPK inhibition; IGF-1, mTOR, ERK decrease; reduction in glucose, insulin, and leptin levels	Mitsuhashi et al. 2014, [39]
Endometrial cancer	—	Ishikawa endometrial cancer cells	—	induction of cell cycle arrest, autophagy, apoptosis	Takahashi et al. 2014, [40]
Esophageal adenocarcinoma	—	OE19, OE33, SK-GT4, OACM 5.1C	nude mice	proliferation inhibition; cyclin D1, Cdk4, Cdk6 (cyclin-dependent kinase), EGFR, IGF-1 decrease	Fujihara et al. 2015, [41]
Esophageal cancer	5-fluorouracil	FLO-1, BE3, SKGT-4, OE33, JHESO, OACP, YES-6, KATO-TN	xenograft mice	CSC targeting; PI3K/mTOR signaling pathway inhibition; chemosensitization	Honjo et al. 2014, [42]
Esophageal squamous cell carcinoma	—	EC109, EC9706	nude mice	inhibition of STAT3/Bcl-2; induction of autophagy and apoptosis	Feng et al. 2014, [43]
Esophageal squamous cell carcinoma	—	EC109, EC9706	nude mice	inhibition of cell proliferation and tumor growth; activation of AMPK; induction of cell cycle arrest; upregulation of p53, p21^CIP1^, and p27^KIP1^	Cai et al. 2015, [44]
Fibrosarcoma	—	BHK-21/C13	Syrian golden hamsters	inhibition of fibrosarcoma growth	Popović D.J. et al. 2017, [45]
Fibrosarcoma	caffeine	BHK-21/C13	Syrian golden hamsters	inhibition of fibrosarcoma growth	Popović D.J. et al. 2018, [46]
Fibrosarcoma	itraconazole	BHK-21/C13; MRC-5, BHK-21/C13, HeLa, HT-29, A549	Syrian golden hamsters	inhibition of fibrosarcoma growth	Popović K.J. et al. 2019, [47]
Fibrosarcoma	nitroglycerin	BHK-21/C13	Syrian golden hamsters	inhibition of fibrosarcoma growth, HIF-1α and downstream targets (VEGF, P-pg-MDRP, MRPs)	Popović K.J. et al. 2020, [48]
Fibrosarcoma	disulfiram	BHK-21/C13	Syrian golden hamsters	inhibition of fibrosarcoma growth, NF-κB, HIF-1α, and downstream targets (VEGF, P-gp, and MRPs)	Popović K.J. et al. 2021, [49]
Fibrosarcoma	diclofenac, caffeine, itraconazole, nitroglycerin, disulfiram, deoxycholic acid	BHK-21/C13	Syrian golden hamsters	reduction in mutational status, tumor proliferation, neoangiogenesis, and glucose/NO metabolism; modulation of apoptosis in correlation with tumor size; inhibition of fibrosarcoma growth	Popovıć J.K. et al. 2024, [50]
Fibrosarcoma	caffeine, itraconazole, nitroglycerin	BHK-21/C13	Syrian golden hamsters	inhibition of fibrosarcoma growth	Popovıć D.J. et al. 2024, [51]
Fibrosarcoma	disulfiram, deoxycholic acid	BHK-21/C13	Syrian golden hamsters	inhibition of fibrosarcoma growth and MAPK/PI3K/AKT/ /mTOR/NF-κB signaling pathways	Popovıć K.J. et al. 2024, [52]
Gastric cancer	—	SGC7901, BGC-823	patient tissues	HIF1α/PKM2 signaling inhibition, cancer cell viability decrease, induction of apoptosis and cell cycle arrest; PI3K, Akt, HIF1α, PARP, PKM2 and COX decrease	Chen et al. 2015, [53]
Gastric Cancer	—	MKN1, MKN45, KATO-III, SNU-1	nude mice	AMPK and NF-ĸB pathway activation; cell proliferation inhibition; peritoneal metastasis suppression	Sekino et al. 2018, [54]
Glioblastoma	temozolomide and/or irradiation	U87 (ATCC HTB-14), LN18 (ATCC CRL-2610), U251, SF767	nude mice	cell growth inhibition; reduction in mitochondrial-dependent ATP production and proliferation; induction of cell cycle arrest, autophagy, apoptosis, and cell death; sensitization to temozolomide and/or irradiation therapy	Sesen et al. 2015, [55]
Glioblastoma	—	U87-MG, T98G	mice	tumor growth suppression; cancer cells growth, viability, and invasiveness inhibition; AMPK/FoxO3a/survivin pathway activation	Cavaliere et al. 2026, [56]
Head and neck cancer (hypopharyngeal squamous cell carcinoma)	—	FaDu HNSCC	mice	inhibition of tumor oxygenation, metabolism, and growth; reduction in Ki-67 proliferation marker expression	Verma et al. 2018, [57]
Head and neck squamous cell carcinoma	—	UMSCC-47s, Cal27s	patient tumor tissue and blood samples	activation of natural killer (NK) cells; upregulation of pSTAT1; downregulation of pSTAT3; inhibition of CXCL1	Crist et al. 2022, [58]
Head and neck squamous cell carcinoma (HNSCC)	—	CAL27, CAL33, UMSCC47	nude mice	prevention of HNSCC cancer stem cell (CSC) progression	Wu et al. 2019, [59]
Hepatocellular carcinoma	radiation	Huh7, HepG2, Hep3B	—	radiosensitization; AMPK activation; DNA damage enhancement	Kim et al. 2014, [60]
Hepatocellular carcinoma	sorafenib	MHCC97H	nude mice	tumor suppressor TIP30 increase; proliferation inhibition; lung metastasis reduction	Guo et al. 2016, [61]
Hepatocellular carcinoma	—	HepG2, Huh7	nude mice	HIF-1α inhibition; mitochondrial oxygen consumption suppression; tumor growth delay	Zhou et al. 2016, [62]
Hepatocellular carcinoma	—	HepG2	—	tumor cell proliferation inhibition; AMPK/mTOR pathway activation	Lv et al. 2018, [63]
Hepatocellular carcinoma	—	HepG2, Huh-7, LX-2, HeLa, HEK293	—	enhancement of AMPK-mediated autophagy-related cancer cell death via metformin binding to VDAC1; AMPK pathway activation; mTOR inhibition; reduction in mitochondrial ROS (mROS)	Ko et al. 2024, [64]
Hepatocellular carcinoma	—	HepG2, LO2	—	enhancement of G3P and glucose-derived glycerol production; inhibition of glucose incorporation into lactate, alanine, glutamate, and glycine	Teng et al. 2024, [65]
Leukemia (chronic lymphocytic leukemia, CLL)	ritonavir	CLL cells from HIV patients	HIV patients	sensitization to ritonavir therapy	Adekola et al. 2015, [66]
Leukemia (chronic lymphocytic leukemia)	fludarabine, ABT-737	cells from the peripheral blood of CLL patients	patients’ peripheral blood	induction of apoptosis; cell cycle inhibition; Mcl-1 downregulation	Bruno et al. 2015, [67]
Li–Fraumeni syndrome (cancer predisposition disorder)	—	HCT116	mice	inhibition of mitochondrial respiration and activity; activation of antiproliferative signaling	Wang et al. 2017, [68]
Lung adenocarcinoma	—	A549, HCC827	nude mice	STAT3 and IL-6 inhibition, AMPK activation	Zhao et al. 2014, [69]
Lung cancer	—	Calu-1 (NSCLC)	—	direct hexokinase-II inhibition; ↓glycolysis	Salani et al. 2013, [70]
Lung cancer	doxorubicin	A549, NIH-3		apoptosis increase; tumor growth inhibition; sensitization to doxorubicin therapy	Nazemiyeh et al. 2025, [71]
Lung cancer: non-small-cell lung cancer (NSCLC); colon cancer	—	H1299, HCT116	—	inhibition of cell proliferation; reduction in mitochondrial-dependent metabolic intermediates required for cell growth; dose-dependent reduction in ATP levels	Griss et al. 2015, [72]
Lung cancer: non-small-cell lung cancer (NSCLC), lung adenocarcinoma, lung squamous cancer, large-cell lung cancer	—	NCI-H522, NCI-H2342, NCI-H2405, NCI-A549, SPC-A-1, SW900, NCI-H1869, SK-MES-1, NCI-H661, NCI-H1299, NCI-H1581, BEAS-2B	patient lung tissue samples, nude mice	inhibition of Nemo-like kinase (NLK), NSCLC cell proliferation and stemness	Suwei et al. 2015, [73]
Lung cancer: non-small-cell lung cancer (NSCLC)	epigallocatechin-3-gallate (EGCG)	A549, H1299, H460, BEAS-2B	nude mice	induction of ROS production; suppression of Nrf2/HO-1 signaling pathway; inhibition of tumor growth; sensitization to EGCG treatment	Yu et al. 2017, [74]
Lung cancer: non-small-cell lung cancer (NSCLC)	—	2B (BEAS-2B) and malignant (1170) derivative, H522, H2030, H1299, H2009, H838, A549, H1975	mice	induction of cell cycle arrest; upregulation of mitochondrial ROS (mROS) and NRF2; reduction in tumor size	Shameem et al. 2023, [75]
Lung cancer: small-cell lung cancer (SCLC)	cisplatin	H446, H526, H446/DDP, H526/DDP	nude mice	suppression of cancer cell growth, EGFR/AKT and mTOR signaling; induction of autophagy, apoptosis, cell cycle arrest, and AMPK; sensitization to cisplatin therapy and alleviation of its toxicity	Xia et al. 2025, [76]
Melanoma	—	B16F10	—	dose-dependent inhibition of cell motility, invasion, and migration; upregulation of E-cadherin	Liang et al. 2015, [77]
Multiple myeloma, breast, melanoma, and ovarian cancer	ritonavir	KMS11, L363, JJN3, NCI-60	patient samples, mice	suppression of Mcl-1 (Bcl-2 family member), AKT, AMPK and mTORC1; induction of apoptosis	Dalva-Aydemir et al. 2015, [78]
Nasopharyngeal carcinoma	radiation	CNE-2, HONE-1, SUNE-1	—	inhibition of DNA repair pathways; radiosensitization	Li et al. 2014, [79]
Oral squamous carcinoma	—	CAL27 (ATCC), CAL33, UMSCC47	nude mice	inhibition of cell proliferation, mTOR signaling, and tumor growth	Madera et al. 2015, [80]
Osteosarcoma	—	MG63	—	inhibition of proliferation, metastasis, and CSC formation, self-renewal and differentiation; Oct-4 and Nanog decrease; activation of AMPK/mTOR/S6 signaling pathway	Chen et al. 2015, [81]
Osteosarcoma	—	KHOS/NP, HOS, MG-63, U-2 OS	nude mice	activation of AMPK; suppression of cancer cell proliferation, migration, and mTOR pathway	Ko et al. 2016, [82]
Osteosarcoma	—	OS MG63, U2OS	—	proliferation and metastasis inhibition; Akt inhibition; PTEN increase	Li et al. 2018, [83]
Ovarian cancer	cisplatin	SKOV3, A2780	nude mouse xenografts	CSC targeting; AMPK activation; proliferation inhibition	Shank et al. 2012, [84]
Ovarian cancer	paclitaxel	Ovcar-5, Kras/PTEN, SKOV3ip1, Kuramochi, HeyA8, IOSE	mice	cell cycle arrest; sensitization to paclitaxel therapy; tumor reduction	Lengyel et al. 2015, [85]
Ovarian cancer	cisplatin	SKOV3, A2780	mice	inhibition of cancer cell growth, tumor growth, and cancer stem cell (CSC) self-renewal; sensitization to cisplatin therapy	Zhang et al. 2015, [86]
Ovarian cancer	sirtuin 3 (SIRT3)	SKOV3, A2780, HO8910	—	inhibition of cancer cell growth; energy stress and apoptosis induction; AMPK activation	Wu et al. 2018, [87]
Ovarian cancer	fasting cycles	SKOV3, A2780	nude mice	reduction in cell viability and proliferation under low-glucose conditions; induction of apoptosis and ferroptosis	Wu et al. 2025, [88]
Pancreatic cancer	—	—	Syrian golden hamsters	↓ insulin signaling; inhibition of islet proliferation; prevention of pancreatic carcinogenesis	Schneider et al. 2001, [2]
Pancreatic cancer	—	ASPC-1, BxPC-3, PANC-1, SW1990	—	induction of caspase-dependent apoptosis; ↓EGFR and P-MAPK signaling	Wang et al. 2008, [89]
Pancreatic cancer	—	PANC-1, MIaPaCa-2, BxPC-3	Nude mice xenografts	AMPK activation; ↓mTOR; inhibition of insulin/GPCR receptor signaling crosstalk	Kisfalvi et al. 2009, [90]
Pancreatic cancer	—	Panc1, Panc28, L3.6pL	nude mice	mTOR, AKT and EGFR/Ras inhibition	Nair et al. 2014, [91]
Pancreatic cancer	tested: new mitochondria-targeted metformin analogues	MiaPaCa-2, PANC-1, HPNE, IEC-6, MCF-10A	mice	AMPK activation; cell cycle arrest induction; radiosensitization enhancement	Cheng et al. 2016, [92]
Pancreatic cancer	—	Panc1, PK1, PK9	nude mice	inhibition of cell proliferation and tumor growth; downregulation of cyclin D1, EGFR, and IGF-1R	Kato et al. 2016, [93]
Pancreatic cancer	tested: new class of mitochondria-targeted metformin analogs	MiaPaCa-2, PANC-1	—	inhibition of mitochondrial function; stimulation of ROS production and redox signaling; activation of AMPK; induction of antiproliferative effects	Kalyanaraman et al. 2017, [94]
Pancreatic cancer	—	BxPC-3, PANC-1, SW1990, MIaPaCa-2	nude mice	miR-378a-3p increase; miR-378a-3p /VEGFA/RGC-32 pathway regulation; cancer cell proliferation, invasion, and migration decrease; apoptosis increase	He et al. 2024, [95]
Pancreatic cancer (pancreatic ductal adenocarcinoma)	—	BxPC-3, CFPAC-1, PANC-1, Capan-2, MIaPaCa-2	nude mice	HNF4G degradation; AMPK activation	Wang et al. 2021, [96]
Prostate adenocarcinoma, prostate cancer, melanoma, lung adenocarcinoma	—	LNCaP, DU145, A375, A549, P69	mice	induction of endoplasmic reticulum (ER) stress; stimulation of ER calcium release and mitochondrial calcium uptake; induction of mitochondrial swelling	Loubiere et al. 2017, [97]
Prostate cancer	2-deoxyglucose	LNCaP, P69, PC3, DU145	—	inhibition of glycolysis; induction of metabolic stress and p53-dependent apoptosis	Ben Sahra et al. 2010, [98]
Prostate cancer	—	Vcap, PC-3	—	miR30a increase, SOX4 decrease	Zhang et al. 2014, [99]
Prostate cancer	—	PC-3, LNCaP	mice	IGF-1, ERK and Akt decrease; tumor growth reduction	Kato et al. 2015, [100]
Prostate cancer	—	LNCaP, DU145, PC3	—	lipogenesis inhibition, dose-dependent ATP decrease	Loubiere et al. 2015, [101]
Prostate cancer	—	PC3, PNT1A	—	activation of AMPK; suppression of cancer cell proliferation, migration, and ERK1/2 signaling	Landim et al. 2018, [102]
Prostate cancer	enzalutamide	LNCaP, MR49F, C4–2, C4–2R, 22Rv1, LuCaP35CR	nude mice	suppression of cancer cell proliferation and viability; inhibition of mitogen-activated protein kinase (MAPK) signaling; overcoming of enzalutamide resistance	Simpson et al. 2025, [103]
Prostate cancer, cervical cancer, ovarian cancer	—	PC3, HeLa S3, OVCAR3	—	AMPK activation; induction of protein acetylation	Galdieri et al. 2016, [104]
Prostate cancer; breast cancer; lung cancer	—	LNCaP, DU145, PC3, REDD1^−/−^ and p53^−/−^ mouse embryonic fibroblasts; MCF7; A549	—	p53-dependent upregulation of REDD1 expression; REDD1-mediated mTOR inhibition; AMPK-independent induction of cell cycle arrest	Ben Sahra et al. 2011, [105]
Renal cancer	—	786-O	—	upregulation of microRNA-26a and PTEN; downregulation of Bcl-2 and cyclin D1; inhibition of renal cancer cell growth	Yang et al. 2014, [106]
Salivary adenocarcinoma	pp242 (an mTOR inhibitor)	HSY, HSG	nude mice	suppression of cell growth, proliferation, and MYC expression; induction of cell cycle arrest and apoptosis; restoration of P53; enhancement of tumor necrosis	Guo et al. 2015, [107]
Thyroid cancer	2-deoxy-D-glucose	FTC133, BCPAP	patients’ tissue samples	inhibition of cell proliferation; induction of endoplasmic reticulum (ER) stress, autophagy, and cell death	Bikas et al. 2015, [108]
Thyroid cancer	—	SW579, TT	nude mice	inhibition of mTOR pathway; downregulation of c-Myc and cyclin D1; suppression of cancer cell growth, proliferation, and migration	Han et al. 2015, [109]
Thyroid cancer	—	FTC133, BCPAP	mice	inhibition of mitochondrial glycerophosphate dehydrogenase (MGPDH) and tumor growth	Thakur et al. 2018, [110]
Thyroid carcinoma	chemotherapeutic agents (doxorubicin, cisplatin)	HTh74 HTh74Rdox, SW1736, C643, FTC133, FRTL-5	—	activation of AMPK; induction of apoptosis; inhibition of cancer stem cell (CSC) self-renewal	Chen et al. 2012, [111]
Uterine leiomyoma	—	ELT-3 (rat-derived uterine leiomyoma cells)	—	inhibition of VEGF and mTORC1/HIF-1α pathway	Tadakawa et al. 2015, [112]

* Note: To ensure absolute fidelity to the primary literature and facilitate accurate cross-referencing, all cell line designations, genes, and protein nomenclatures have been retained exactly as reported in the original cited source publications.

## Appendix B

The interconnected regulatory network involving AMPK, Akt, mTOR, and NF-κB is summarized in Appendix B based on the literature review [127]. Effects on downstream targets are denoted as stimulation (/), while inhibitory interactions are indicated by (\).

- \ mTOR \ NF-κB;
- \ mTOR, TLR4, NF-κB /ATG;
- Akt / mTOR / NF-κB;
- Akt / iNOS, VEGF, HIF-1α, HIF-2α, mTOR, NF-κB;
- Akt / mTOR / HIF / IκBα/ NF-κB / HIF / VEGF;
- ATG (ATG5) \ NF-κB, mTOR;
- Beclin1, ATG3, ATG4D, ATG5 / mTOR \autophagy;
- CD31 / Akt / mTOR / HIF / IκBα / NF-κB / HIF / VEGF;
- EGF, VEGF, PI3K, Akt, JAK2, ERK, mTOR / PAK1 / Raf, MEK1, Cyclin D_1_, Cyclin B_1_, RAS, MAPK, HIFs, p38, PI3K, Akt, Beclin-1, mTOR, ROS, Wnt/β-catenin, JNK, TLR4, NF-κB, ATG5, ATG12, ATG16L, TGF-β, STAT5;
- EGF / VEGFR / Akt / mTOR / HIF / IκBα / NF-κB / HIF / VEGF; VEGF / VEGFR / PKC / iNOS / NO;
- EGFR, Raf, RAS, p38 (MAPK), MEK, ERK, Akt, JNK, IGF, mTOR, TGF-α, STAT3, PI3K, PKC / IκBα / NF-κB / MDR (MDPRs, ABC transporters), P-gp;
- EGFR / PI3K / Akt / mTOR / IκBα / NF-κB / P-gp (P-gp = MDR1 = ABCB1);
- EGFR / Raf / RAS / p38 (MAPK) / MEK / ERK / Akt / JNK, mTOR / IκBα / NF-κB / P-gp;
- IGF-1, IGF-2 / mTOR / IκB / NF-κB / P-gp;
- IKK / AMPK \ mTOR / JNK, p53, Bcl-2, Beclin1, PAK1 / autophagy;
- IKK \ IκB; RTKs / PI3K / Akt / mTOR;
- JAK / STAT / PI3K / Akt / mTOR / NF-κB;
- MEK / ERK / Akt / mTOR, JNK, HIF, iNOS, VEGF / IκBα / NF-κB / …;
- mTOR / HIF-1α;
- MYC / Cyclins, mTOR, Akt, Oct4, Sox2, Klf4, Nrf1, HIFs, CDK4, STATs;
- PCNA / p38MAPK / JNK / ERK / PI3K / Akt / mTOR / IκBα / NF-κB / P-gp;
- PI3K, mTOR \ p21, p27;
- PI3K, PKB, Akt, IGF, NF-κB / mTOR \autophagy;
- PI3K / Akt / mTOR / HIFs / VEGF, NF-κB;
- PTEN \ PI3K / Akt / mTOR / NF-κB;
- PTEN\JAK / STAT3 / PI3K / Akt / mTOR / IκBα / NF-κB;
- TGF-α, STAT3, p38 MAPK, MEK, ERK, EGFR, Raf, RAS / PI3K / Akt, mTOR, JNK, PKC, p38 MAPK / IκBα / NF-κB / HIF / VEGF; PTEN \ PI3K;
- TGF-α / PI3K / Akt / mTOR / HIF / IκBα / NF-κB / HIF / VEGF;
- TGF-α / PI3K / Akt / mTOR / IκB / NF-κB / P-gp (MDR1-ABCB1);
- TNF, ROS / (NF-κB / HIF-1α), (NF-κB \ p53), mTOR \ autophagy;
- TNF, ROS / NF-κB, HIF1α, mTOR \autophagy (ATG), p53, apoptosis;
- VEGF / VEGFR / NF-κB / VEGF;
- VEGF / VEGFR / PKC / RAS / Raf1 / MEK (MAP2 / ERK kinase) / ERK(1, 2) / Akt / mTOR / IκBα / NF-κB / …;
- VEGF / FGF / PDGF / RTKs / PI3K / AKT / mTOR.
